# Risk Prediction in Patients With Metabolic Dysfunction–Associated Steatohepatitis Using Natural Language Processing

**DOI:** 10.1016/j.gastha.2025.100701

**Published:** 2025-05-14

**Authors:** Jordan Guillot, Christopher Y.K. Williams, Shadera Azzam, Balu Bhasuran, Gail Fernandes, Boshu Ru, Joe Yang, Xiao Zhang, R. Ravi Shankar, Jin Ge, Vivek A. Rudrapatna

**Affiliations:** 1 Bakar Computational Health Sciences Institute, University of California, San Francisco, California; 2 Merck & Co., Inc., Rahway, New Jersey; 3 Division of Gastroenterology, Department of Medicine, University of California, San Francisco, California

**Keywords:** Real-World Evidence, Natural Language Processing, Metabolic Dysfunction–Associated Steatotic Liver Disease, Cirrhosis

## Abstract

**Background and Aims:**

Metabolic dysfunction–associated steatohepatitis (MASH) is a highly heterogenous condition and a leading cause of end-stage liver disease. Understanding disease progression in real-world settings remains a major unmet need. We sought to define a real-world MASH cohort using natural language processing (NLP) and identify significant associations with all-cause mortality and progression to cirrhosis and liver transplantation.

**Methods:**

We developed, validated, and applied a novel NLP algorithm, “NASHDetection,” to identify patients at the University of California San Francisco who were diagnosed with MASH between 2012 and 2022. We used Cox regression with bidirectional stepwise variable selection to identify significant associations with outcomes.

**Results:**

NASHDetection was 86% accurate at identifying 2695 MASH patients. At the time of their diagnosis, the median age was 57 years; 55.4% had cirrhosis at baseline, with 34.0% having evidence of decompensation and 10.8% with hepatocellular carcinoma. The most common comorbidities were hypertension (61.9%), hyperlipidemia (47.4%), and type 2 diabetes mellitus (41.5%). Multiple comorbidities were associated with all-cause mortality, including type 2 diabetes mellitus (hazard ratio (HR): 1.36; confidence interval (CI): 1.07–1.73), heart failure (HR: 1.45; CI: 1.01–2.08), and peripheral artery disease (HR: 1.72; CI: 1.04–2.85). Significant laboratory-based predictors of mortality included high–low-density lipoprotein cholesterol (HR: 1.49; CI: 1.20–1.84) and high alkaline phosphatase (HR: 1.94; CI: 1.58–2.38).

**Conclusion:**

We described a cohort of real-world MASH patients using a new NLP algorithm and found several potential predictors of progression to all-cause mortality, cirrhosis, and liver transplantation. The use of NLP to characterize these patients can help support the development of future interventional trials in MASH.

## Introduction

With the increasing prevalence of metabolic syndrome worldwide, metabolic dysfunction–associated steatotic liver disease (MASLD) is now one of the leading causes of end-stage liver disease, affecting up to 25% of the global population.^1^ MASLD is a heterogenous condition with wide variations in clinical presentation that are thought to be driven by demographics, genetics, comorbid clinical conditions, microbiota exposures, and metabolic milieu.^2^ There is increasing recognition that social determinants of health, such as socioeconomic levels, educational attainment, and food insecurity are also associated with the natural history of MASLD.^3^ Much of our current knowledge about the trajectories of patients with MASLD and metabolic dysfunction–associated steatohepatitis (MASH), however, has come from curated research registries, providing significant insights albeit with unclear generalizability and scalability.^4^ Therefore, understanding disease progression and identifying potential opportunities for intervention in real-world settings remain a major unmet need.

Studying patients with MASH using electronic health records (EHR) data presents several challenges. While International Classification of Diseases (ICD) codes are commonly used to define retrospective research cohorts, they have well-known limitations and inaccuracies. Procedure codes such as liver biopsy, once the primary method for diagnosing MASH, as well as newer and less invasive methods like magnetic resonance elastography and transient elastography (eg, Fibroscan), nowadays recommended to establish diagnosis, may be used^5^; however, these procedures alone could be insufficient to make a MASH diagnosis and the guidelines have been changing over the past decade. Furthermore, the results and interpretation of these tests are commonly captured as free-text reports rather than as structured data and many patients receive care across multiple health systems, increasing the risk of missing codes within a single EHR database. Physician notes, often considered the gold standard for ascertaining MASH diagnoses, account for all sources of clinical data including those that are not searchable in EHR databases and incorporate clinical reasoning pertaining to alternative diagnoses. Indeed, recent studies have shown that using natural language processing (NLP) to ascertain clinical diagnoses directly from these notes can surpass ICD code–based methods at identifying patients with MASLD.^6^^,^^7^ However, algorithms that specifically identify those with MASH have not yet been validated.

We developed a new algorithm that identifies MASH cases based on clinical assessments made by hepatologists and applied it to the University of California San Francisco (UCSF) EHR data to identify a real-world MASH cohort and characterized patient trajectories following diagnosis. Our primary objective was to identify predictors of all-cause mortality. Our secondary objectives were to examine associations of clinical predictors with (1) progression to cirrhosis in MASH patients without cirrhosis and (2) progression to liver transplantation in MASH patient with cirrhosis.

## Methods

### Data Source and Study Population

We used the UCSF deidentified clinical data warehouse to develop an NLP-based algorithm for identifying MASH cases, called NASHDetection. We identified notes associated with a hepatology or gastroenterology encounter at UCSF between January 2012 and December 2022. NASHDetection incorporates a set of MASH-related keywords to identify potential cases, and uses negation phrases for exclusion (Supplementary Methods, code available at github.com/rwelab/NASHDetection).

To validate the algorithm, 300 notes were randomly sampled to generate the development (n = 150) and test (n = 150) sets, which were independently labeled for the presence or absence of a MASH diagnosis by 2 independent reviewers (C.Y.K.W. and S.A.). Disagreements were resolved by a third review (J.Ge.). NASHDetection was refined based on its performance on the development set. The hold-out test set was used once to assess model performance. This achieved a sensitivity of 89%, specificity of 84%, accuracy of 86%, and F1 score of 0.84.

### Inclusion and Exclusion Criteria

**Figure 1:**
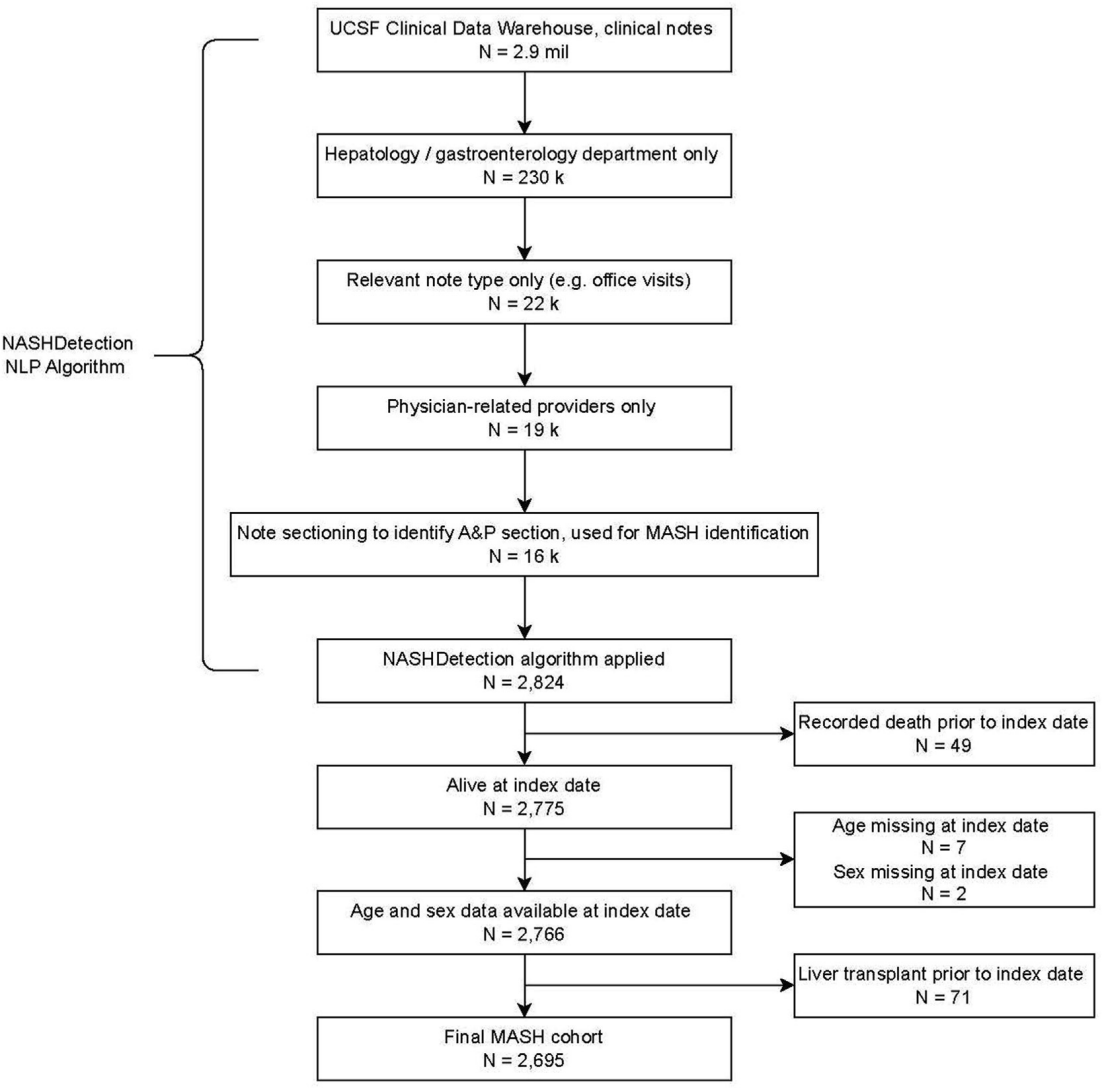
NASHDetection NLP methodology utilizing notes from the clinical data warehouse using named entity recognition and regular expressions, MASH assertions were identified in the A&P of the clinical encounter. After validation, NASHDetection was applied to the entire corpus of 58,994 notes (referred as ‘Note sectioning to identify A&P section’) for classification of presence/absence of MASH; the earliest note for each patient marked as positive for MASH was taken as the 'index note' from which the 'index date' was derived. A&P, assessment and plan.

To establish the cohort, we applied NASHDetection to all adult hepatology or gastroenterology encounter notes at UCSF (58,994 notes; Figure 1). The index date (baseline) was defined as 90 days after the earliest date at which NASHDetection identified a MASH case. This corresponds to the date at which this diagnosis is made or recognized by a specialist at UCSF, adding 90 days to allow for delayed recording in clinical notes (see Figure A1).

Patients were excluded if there was a death or liver transplant record (using CPT coding) before the index date or the age or sex was missing from the record. We excluded patients diagnosed with MASH after orthotopic liver transplantation, as these patients are clinically distinct from patients with MASH in their native livers.^8^ We defined 2 subcohorts: those with cirrhosis at baseline and those without cirrhosis at baseline using ICD coding.

### Variables and Outcomes

Fifty-eight demographic and clinical features (Supplementary Data) thought to be clinically relevant, widely available in the EHR, and explored in previous studies for predicting MASH progression were selected as covariates. The primary outcome was all-cause mortality. The primary source of information about patient death was the California state death registry, which is linked to the EHR database. The secondary outcomes were (1) incident cirrhosis defined as any diagnosis of cirrhosis (ICD codes) and (2) liver transplant defined as any transplant procedure using CPT code 47135. The occurrence of cirrhosis was studied in the noncirrhosis subcohort and the occurrence of liver transplant was studied in the cirrhosis subcohort. Follow-up for primary and secondary outcomes was conducted from the index date through December 31, 2023. Clinical features and outcomes were extracted and curated from the EHR (Supplementary Methods).

### Statistical Analyses

Baseline characteristics were described using medians and interquartile range (IQR; presented as 25th–75th percentiles) for continuous variables and percentages for categorical variables. Hazard ratios (HRs) and 95% confidence intervals (CIs) associated with baseline extracted features were estimated using Cox proportional hazards models. We imputed missing values using random forest models, as implemented by the MICE package in R. For each of the primary and secondary outcomes, patients with an outcome recorded before the index date (ie, 90 days after the within-system MASH diagnosis date) were excluded from the Cox model to account for delayed coding in the EHR. In competing risk analyses, patients were censored if they experienced such competing events at a date before 3 months before the outcome of interest was recorded. We used Cox proportional hazards regression to estimate the cause-specific HR for the chosen predictors and all-cause mortality and cirrhosis and liver transplant in the corresponding subcohorts.

All modeling was initially conducted on the full set of selected predictors. To improve the interpretability of the model, we performed bidirectional stepwise variable selection to sequentially remove nonsignificant predictors or added predictors that would improve stability of the model (Supplementary Methods).

For the bidirectional stepwise variable selection, we followed the following steps: (1) Remove the variable with the highest *P* value. (2) If the Akaike information criterion (AIC) decreased, the variable was removed from the model; if the AIC increased, the variable was added into the model. (3) The previously removed variables were one by one readded in the model. (4) If the AIC increased, the variable was removed again, if the AIC decreased the variable will be readded in the model. (5). Repeat steps 1, 2, 3, and 4 until reaching variables with a *P* value ≤ .05.

The following variables were kept in all models and not subject to variable selection: age, sex, race/ethnicity and calculated baseline Model for End-Stage Liver Disease (MELD) 3.0 scores. This was done because of their a priori importance, and to help stabilize the estimates of other variables. All significant results for select outcomes are presented as HR (95% CI). See Supplementary Materials for tables of full results for the primary and secondary outcomes.

All data and queries were extracted, processed, and analyzed using PySpark, Python 3.8.17, and R. This study was approved by the UCSF Institutional Review Board under Study# 21–33854.

## Results

### Patient Baseline Characteristics

Using NASHDetection, we identified 2824 patients who were diagnosed with MASH at UCSF between 2012 and 2022. We then excluded 129 patients due to death or liver transplantation occurring before index date or due to missing age or sex. All patients had a defined race/ethnicity. A total of 2695 MASH patients were included in the final cohort. Among them, 1494 (55.4%) were identified to have baseline cirrhosis (decompensated or compensated) and constitute the cirrhosis subcohort, leaving 1201 patients in the noncirrhosis subcohort. Detailed characteristics of the cohorts are reported in Table 1.

**Table 1:** Total MASH Cohort and Subcohort Characteristics

Baseline characteristics	MASH cohort N = 2695	Cirrhosis subcohort N = 1494	Noncirrhosis subcohort N = 1201
Sex			
Female	1446 (53.7%)	796 (53.3%)	650 (54.1%)
Male	1249 (46.3%)	698 (46.7%)	551 (45.9%)
Ethnicity/Race			
White	1003 (37.2%)	550 (36.8%)	453 (37.7%)
Hispanic/Latino	860 (31.9%)	562 (37.6%)	298 (24.8%)
Asian	427 (15.8%)	156 (10.4%)	271 (22.6%)
Native American/Alaskan/Hawaiian or other	352 (13.1%)	196 (13.1%)	156 (13.0%)
Black/African American	53 (2.0%)	30 (2.0%)	23 (1.9%)
Area Deprivation Index			
Median national	12.4 [4.0–32.3]	19.6 [6.0–41.0]	5.2 [2.4–19.5]
Median state	4.6 [2.1–8.1]	6.4 [2.9–8.8]	2.5 [1.5–6.4]
Insurance			
Commercial	1216 (45.1%)	532 (35.6%)	684 (57.0%)
Medicare	836 (31.0%)	598 (40.0%)	238 (19.8%)
Medicaid	592 (22.0.%)	355 (23.8%)	237 (19.7%)
Self-pay or other	51 (1.9%)	9 (0.6%)	42 (3.5%)
Age at time of within system diagnosis			
Median age (y)	57 [45–65]	61 [53–67]	50 [37–60]
18–40	448 (16.6%)	89 (6.0%)	359 (29.9%)
41–60	1093 (40.6%)	566 (37.9%)	527 (43.9%)
61–65	447 (16.6%)	320 (21.4%)	127 (10.6%)
> 65	707 (26.2%)	519 (34.7%)	188 (15.7%)
Year of within system MASH diagnosis			
Median year	2018 [2016–2021]	2018 [2016–2021]	2018 [2016–2021]
Before 2020	1726 (64.0%)	953 (63.8%)	773 (64.4%)
After 2020 (COVID era)	969 (36.0%)	541 (36.2%)	428 (35.6%)
Body mass index (BMI)			
Median BMI (kg/m^2^)	30.7 [26.7–35.4]	31.0 [26.9–36.0]	30.6 [26.5–34.7]
BMI categories^a^			
Underweight	11 (0.4%)	6 (0.4%)	5 (0.4%)
Normal	255 (9.5%)	152 (10.2%)	103 (8.6%)
Overweight	688 (25.5%)	379 (25.4%)	309 (25.7%)
Obese	1349 (50.1%)	762 (51.0%)	587 (48.9%)
Missing	392 (14.5%)	195 (13.1%)	197 (16.4%)
Cirrhosis status			
Any cirrhosis	1494 (55.4%)	1494 (100.0%)	0 (0%)
Decompensated	917 (34.0%)	917 (61.4%)	0 (0%)
Complications			
Encephalopathy	423 (15.7%)	423 (28.3%)	0 (0%)
Ascites	601 (22.3%)	601 (40.2%)	0 (0%)
Esophageal varices	506 (18.8%)	506 (33.9%)	0 (0%)
Hepatopulmonary syndrome	21 (0.8%)	21 (1.4%)	0 (0%)
Hepatorenal syndrome	37 (1.4%)	37 (2.5%)	0 (0%)
Hepatocellular carcinoma	292 (10.8%)	269 (18.0%)	23 (1.9%)
Comorbid diagnoses			
HTN	1668 (61.8%)	1145 (76.6%)	523 (43.5%)
HLD	1277 (47.4%)	606 (40.6%)	671 (55.9%)
T2DM	1118 (41.5%)	786 (52.6%)	332 (27.6%)
Coronary artery disease	277 (10.3%)	193 (12.9%)	84 (7.0%)
Peripheral artery disease	46 (1.7%)	31 (2.1%)	15 (1.2%)
Obstructive sleep apnea	373 (13.8%)	181 (12.1%)	192 (16.0%)
CKD	295 (10.9%)	223 (14.9%)	72 (6.0%)
HF	124 (4.6%)	87 (5.8%)	37 (3.1%)
Bariatric surgery history	64 (2.4%)	47 (3.1%)	17 (1.4%)
CCI	3 [1–5]	5 [3–6]	1 [1–2]
Medication use			
Aspirin	326 (12.1%)	209 (14.0%)	117 (9.7%)
Statin	654 (24.3%)	400 (26.8%)	254 (21.1%)
Insulin	483 (17.9%)	398 (26.6%)	85 (7.1%)
Metformin	545 (20.2%)	330 (22.1%)	215 (17.9%)
Sulfonylurea	260 (9.6%)	186 (12.4%)	74 (6.2%)
Thiazolidinedione	30 (1.1%)	21 (1.4%)	9 (0.7%)
GLP-1 receptor agonist	114 (4.2%)	68 (4.6%)	46 (3.8%)
DPP-4 inhibitor	105 (3.9%)	71 (4.8%)	34 (2.8%)
SGLT-2 inhibitor	80 (3.0%)	56 (3.7%)	24 (2.0%)
ACE inhibitor	388 (14.4%)	236 (15.8%)	152 (12.7%)
Angiotensin receptor blocker	316 (11.7%)	181 (12.1%)	135 (11.2%)
Diuretic	284 (10.5%)	149 (10.0%)	135 (11.2%)
Calcium channel blocker	326 (12.1%)	180 (12.0%)	146 (12.2%)
Vasodilator	112 (4.2%)	72 (4.8%)	40 (3.3%)
Beta blocker	36 (1.3%)	21 (1.4%)	15 (1.2%)
Alpha blocker	39 (1.4%)	20 (1.3%)	19 (1.6%)
Central acting agent	42 (1.6%)	22 (1.5%)	20 (1.7%)
≥ 2 hypertensive medications	407 (15.1%)	226 (15.1%)	181 (15.1%)

Baseline characteristics	MASH cohort Median [IQR]	Cirrhosis subcohort Median [IQR]	Noncirrhosis subcohort median [IQR]
Lab values/vitals			
MELD-3.0	9.1 [6.4–15.7]	12.5 [8.0–18]	6.3 [5.3–7.5]
Alkaline phosphatase (U/L)	87 [64–123]	108 [78–156]	72 [57–91]
Alanine aminotransferase (U/L)	38 [24–61]	31 [21–48]	49 [30–77]
Aspartate aminotransferase (U/L)	37 [27–53]	40 [29–56]	34 [24–50]
Alpha feto protein (ng/mL)	3.9 [2.6–6.5]	4.2 [2.7–6.9]	3.2 [2.2–4.6]
Total bilirubin (mg/dL)	0.8 [0.5–1.3]	1.1 [0.7–2.1]	0.6 [0.4–0.8]
Platelet count (x10^9^/L)	185 [107–249]	117 [75–184]	236 [193–283]
Albumin (g/dL)	3.9 [3.3–4.2]	3.5 [3–3.9]	4.2 [3.9–4.4]
eGlomerular filtration rate (mL/min)	97 [71–108]	88 [58–105]	103 [89–111]
Creatinine (mg/dL)	0.8 [0.5–1.3]	0.9 [0.7–1.2]	0.8 [0.7–0.9]
Sodium (mEq/L)	138 [136–140]	138 [135–140]	138 [137–140]
INR	1.1 [1–1.3]	1.2 [1.1–1.4]	1 [1–1.1]
Triglycerides (mg/dL)	134 [94–196]	121 [81–178]	141 [101–206]
Low density lipoproteins (mg/dL)	104 [81–132]	94 [70–116]	110 [87–138]
HbA1c (%)	5.7 [5.3–6.6]	5.9 [5.2–7.0]	5.6 [5.3–6.2]
Systolic blood pressure (mm Hg)	129 [117–140]	128 [115–141]	130 [119–140]
Diastolic blood pressure (mm Hg)	72 [63–80]	68 [60–77]	76 [68–83]

DPP-4, dipeptidyl peptidase 4; GLP-1, glucagon-like peptide-1; INR, international normalized ratio; SGLT-2, sodium/glucose cotransporter 2.

a BMI categories were divided between Asian and non-Asian subgroups with Asian BMI categories defined as underweight (<18.5), normal (18.5–22.9), overweight (23–27.4), and obese (≥27.5) and non-Asian BMI categories defined as underweight (<18.5), normal (18.5–24.9), overweight (25–29.9), and obese (≥30).

In the overall MASH cohort, there were 1446 (53.7%) females, with the following race/ethnicities: 1003 (37.2%) White, 860 (31.9%) Hispanic/Latino, 427 (15.8%) Asian, 352 (13.1%) Native American/Alaskan/Hawaiian or other, and 53 (2.0%) Black or African American. The median age at MASH diagnosis was 57 years old and the median body mass index (BMI) was 30.7 kg/m^2^ (IQR [26.7–35.4]). The most common baseline comorbidities included hypertension (HTN; 1668 (61.9%)), hyperlipidemia (HLD; 1277 (47.4%)), type 2 diabetes mellitus (T2DM; 1,118, (41.5%)), obstructive sleep apnea (373 (13.8%)), and chronic kidney disease (CKD; 295 (10.9%)).

Laboratory values at baseline included aspartate aminotransferase (37 U/L (IQR: 27–53)), alanine aminotransferase (38 U/L (24–61)) total bilirubin (0.8 mg/dL (0.5–1.3)), triglycerides (134 mg/dL (94–196)) and HbA1c (5.7% (5.3–6.6)). Baseline MELD 3.0 score was 9.1 (6.4–15.7) in the total cohort with a median score of 6.3 (5.3–7.5) in the noncirrhosis subcohort and 12.5 (8.0–18) in the cirrhosis subcohort. Medication usage included 388 (14.4%) on angiotensin-converting enzyme inhibitors, 316 (11.7%) on angiotensin II receptor blockers, 654 (24.3%) on statins, 545 (20.2%) on metformin, and 483 (17.9%) on insulin.

The cirrhosis subcohort featured a higher proportion of Hispanic/Latino population (37.6% vs 31.9%) and a MASH diagnosis at a later age (61 vs 57). They had a higher proportion of Medicare (40.0% vs 31.0%) and Medicaid (23.8% vs 22.0%) compared to commercial insurance (35.6% vs 45.1%). The cirrhosis subcohort also had a higher percentage of several baseline comorbid conditions, such as HTN (76.6% vs 61.8%), T2DM (52.6% vs 41.5%), and CKD (14.9% vs 10.9%). Lastly, patients in the cirrhosis subcohort exhibited a higher Charlson comorbidity index (CCI; 5 (3–6) vs 3 (1–5)).

#### MASH time-to-event modeling

Among the total cohort, death occurred in 529 patients during follow-up with a 4.4 per 100 person-years incidence. Among the noncirrhosis subcohort, 231 incidents of cirrhosis occurred during follow-up with a 4.5 per person-years incidence. Among the cirrhosis subcohort, 166 liver transplants occurred during follow-up with a 3.1 per 100 person-years incidence (Table 2).

**Table 2:** Total Patients at Risk for the Outcomes of Interest. Event Occurrence Displays the Total Occurrence for That Given Outcome Over the Course of the Study

Clinical event	Total patients at risk for clinical event	Number of events over the study period (2012–2022)	Incidence rate (per 100 PY)	Total PY follow-up
Total cohort to all-cause mortality	2695	529	4.4	11,904.7
No cirrhosis to cirrhosis	1201	231	4.5	5104.4
Cirrhosis to liver transplantation	1494	166	3.1	5341.9

PY, patient year.

### Primary Outcome

#### MASH to all-cause mortality

**Figure 2:**
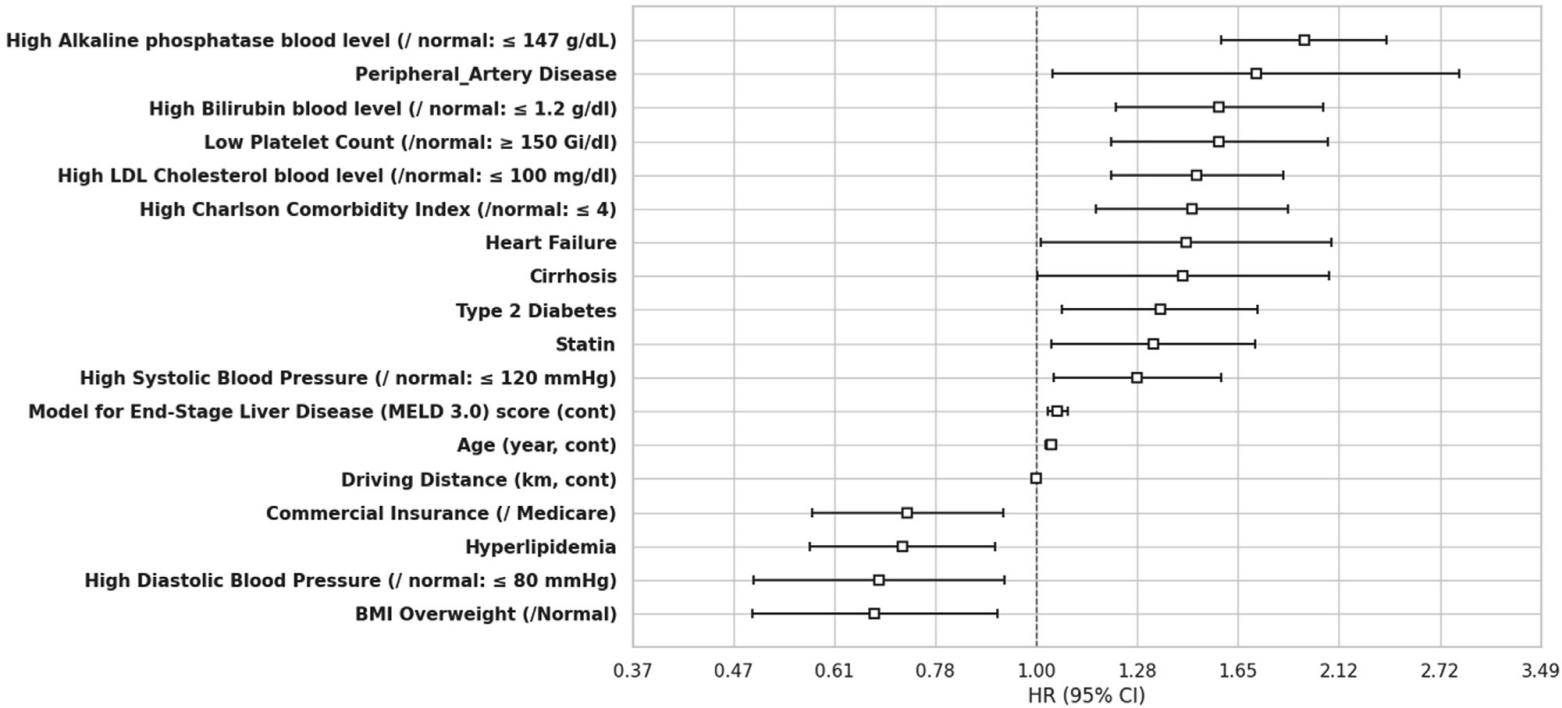
Statistically significant predictors of all-cause mortality. The depicted variables correspond to just those features that were statistically significant in the final Cox regression model subjected to bidirectional stepwise feature selection, with variables of a priori importance enforced for reasons of coefficient stability and interpretation (see methods). For categorical variables, the reference group identified as ‘/reference group.’ All levels of any selected categorical variable were required to be kept in the final model. N patients = 2695/N events = 529.

Nineteen significant covariates are listed in the text below; full results are in the Supplementary Materials. Notably, an overweight BMI (HR: 0.67; 95% CI [0.50–0.91]), high diastolic blood pressure (0.68 (0.50–0.92)), HLD (0.72 (0.57–0.90)), and commercial insurance compared to Medicare (0.73 (0.57–0.92)) were associated with lower all-cause mortality, whereas high systolic blood pressure (1.28 (1.04–1.58)), aspirin users (1.32 (1.00–1.74)) statin users (1.34 (1.04–1.72)), T2DM (1.36 (1.07–1.73)), baseline cirrhosis (1.44 (1.00–2.07)), heart failure (HF) (1.45 (1.01–2.08)), high CCI (1.47 (1.16–1.87)), high–low-density lipoprotein (LDL) cholesterol (1.49 (1.20–1.84)), low platelet count (1.57 (1.20–2.06)), high bilirubin (1.57 (1.22–2.03)), peripheral artery disease (1.72 (1.04–2.85)), and high-alkaline phosphatase (1.94 (1.58–2.38)) were associated with higher all-cause mortality (Figure 2).

### Secondary Outcomes

#### MASH to MASH cirrhosis

**Figure 3:**
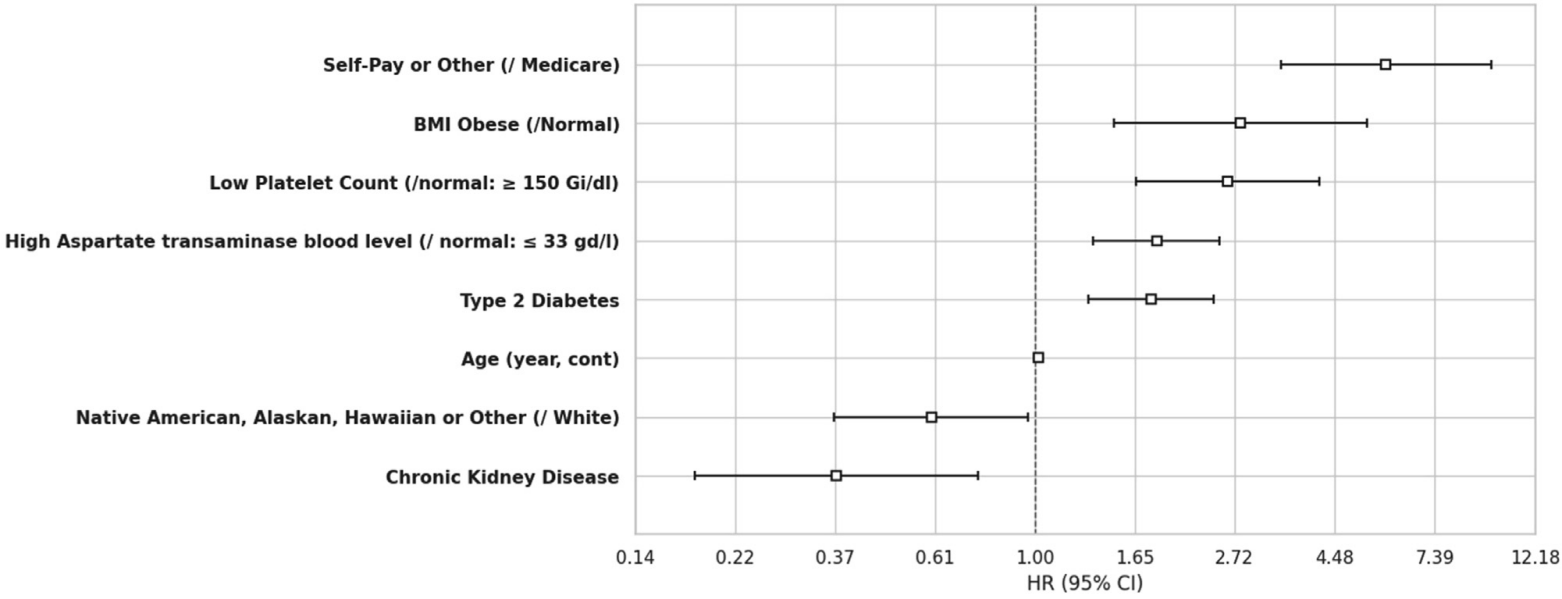
Statistically significant predictors of cirrhosis. The depicted variables correspond to just those features that were statistically significant in the final Cox regression model subjected to bidirectional stepwise feature selection, with variables of a priori importance enforced for reasons of coefficient stability and interpretation (see methods). For categorical variables, the reference group identified as ‘/reference group.’ All levels of any selected categorical variable were required to be kept in the final model. N patients = 1201/N events = 231.

Ten covariates were significantly associated with the development of cirrhosis (compensated and decompensated) with liver transplantation and death acting as competing events. Notably, factors such as CKD (0.37 (0.18–0.75)), Native American, Alaskan, Hawaiian or Other race (0.60 (0.37–0.97)), and HLD (0.74 (0.54–1.01)) were associated with a reduced risk of cirrhosis, whereas factors like T2DM (1.79 (1.31–2.45)), high aspartate transaminase (1.84 (1.34–2.52)), low platelet count (2.62 (1.66–4.14)), high BMI (2.79 (1.48–5.26)), and self-pay or other insurance compared to Medicare (5.78 (3.42–9.79)) were associated with progression to cirrhosis (Figure 3).

#### MASH cirrhosis to liver transplantation

**Figure 4:**
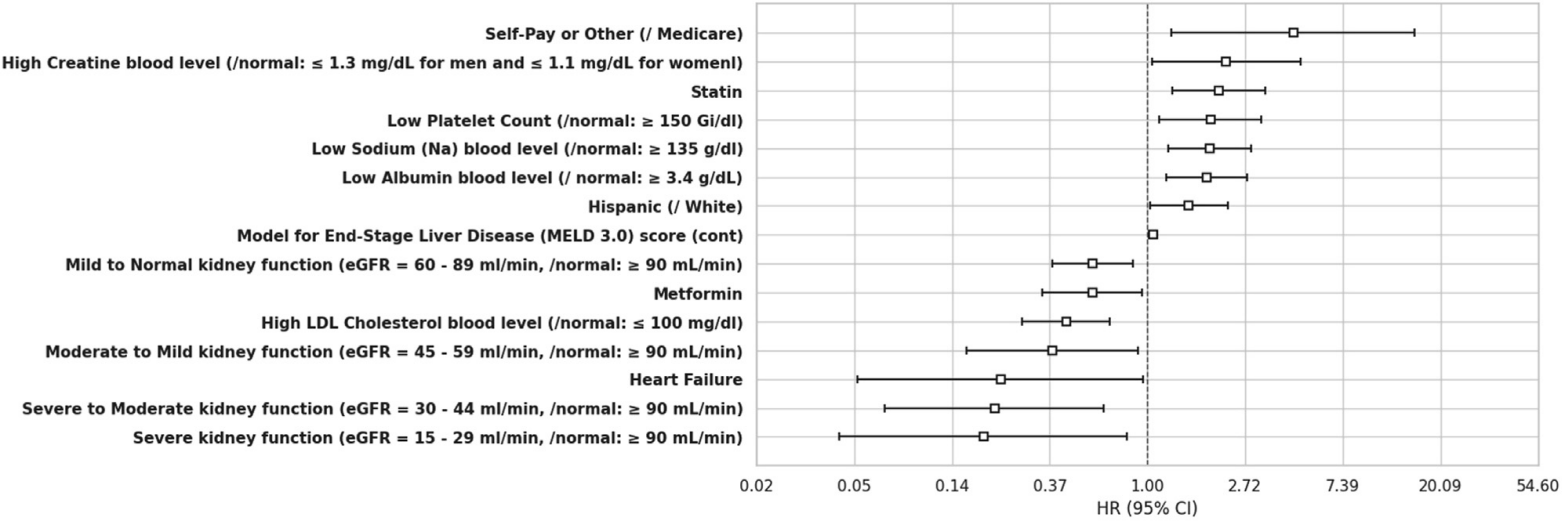
Statistically significant predictors of liver transplantation. The depicted variables correspond to just those features that were statistically significant in the final Cox regression model subjected to bidirectional stepwise feature selection, with variables of a priori importance enforced for reasons of coefficient stability and interpretation (see methods). For categorical variables, the reference group identified as ‘/reference group.’ All levels of any selected categorical variable were required to be kept in the final model. N patients = 1494/N events = 166.

Sixteen covariates were significantly associated with progression to liver transplantation with death acting as a competing event. Notably, factors like severely impaired kidney function (0.19 (0.04–0.81)), moderate to severely impaired kidney function (0.21 (0.07–0.64)), HF (0.22 (0.05–0.96)), mild to moderately impaired kidney function (0.38 (0.16–0.91)), high-LDL cholesterol (0.43 (0.28–0.68)), metformin users (0.57 (0.34–0.94)), mildly impaired kidney function (0.57 (0.38–0.86)), and Medicaid insurance compared to Medicare (0.59 (0.35–1.00)) had lower associations with receiving a liver transplantation, whereas Hispanic ethnicity (1.53 (1.03–2.27)), low albumin (1.83 (1.21–2.78)), low sodium (1.89 (1.24–2.88)), low platelet count (1.90 (1.12–3.22)), statin users (2.07 (1.29–3.32)), high creatinine (2.24 (1.05–4.77)), and self-pay or other insurance (4.43 (1.28–15.37)) had higher associations (Figure 4).

## Discussion

We developed a new NLP algorithm (NASHDetection) to identify a real-world cohort of patients with MASH. By combining this approach with clinical informatics methods and statistical modeling of EHR data, risk factors for progression to cirrhosis, liver transplantation, and all-cause mortality were identified. These risk factors include predictors previously established in the literature, such as T2DM,^9^ as well as new ones like commercial insurance. Taken together, these results lay the groundwork for future studies of this patient population.

A central aspect of our approach involved the use of NLP, given prior studies showing that algorithmic approaches outperform ICD codes alone at accurately identifying cohorts within the spectrum of MASLD.^6^^,^^7^ Our cohort of 2695 patients was diverse along lines of sex, race and ethnicity. It aligned with the demographic profiles seen in real-world cohorts with a slightly higher proportion of Hispanic/Latino patients.^10^ Our cohort’s demographics are likely secondary to the referral patterns observed at UCSF which, as a tertiary referral center for liver transplantation and specialty care, is more reflective of Northern and Central California rather than its immediate vicinity in the San Francisco Bay Area.

A substantial portion of the cohort, 55.4%, presented to UCSF with baseline cirrhosis, with 61.4% already having signs of decompensation. One potential explanation for these findings is that of referral patterns. Many MASH patients are managed in suburban and rural communities in Northern and Central California until they are advanced enough to merit referral for potential transplantation. Another potential explanation is that of delayed diagnoses. Due to the absence of screening guidelines, it is possible that many patients are only diagnosed with MASH after they manifest clear signs of liver decompensation. Future studies assessing these community-based populations during their prediagnosis timeline may help clarify these potential explanations.

The cohort also demonstrated a substantial burden of comorbid conditions, with 61.8% of patients with HTN, 47.4% with HLD, and 41.5% with T2DM. These rates are comparable to a 2020 study of the broader US population of individuals with MASH.^10^ Given these high rates of comorbid conditions, our cohort presented with a moderate CCI score of 3, and thus greater mortality risk, at baseline.^11^ These findings underscore the need for a multidisciplinary approach when managing comorbidities among patients with MASH.

Comorbid T2DM, HF, and peripheral artery disease were associated with all-cause mortality. T2DM has been previously associated with all-cause mortality in patients with MASLD and MASH.^9^ A study published in 2021, identified cardiovascular disease as the leading cause of death in patients with MASLD, mirroring our findings.^12^ Additionally, high LDL cholesterol and high alkaline phosphatase were associated with increased all-cause mortality. Our findings, along with other studies, suggest that metabolic dysfunction severity is associated with worse outcomes and highlight that lab values could serve as signals of progression, encouraging further research to identify and explore potential laboratory results as biomarkers.^13^

Multiple baseline laboratory values were associated with an increased chance of liver transplantation including low albumin, low sodium, low platelet count, and high creatinine. These findings are not surprising as albumin, sodium, and creatinine are relevant components of the MELD score, and low platelet count is often a marker of severe portal hypertension.^14^^,^^15^ Interestingly, in the MASH cirrhosis subcohort, statin use was associated with increased risk of a liver transplantation. This contrasts with previous findings that found that statin users were 63% less likely to undergo liver transplantation compared with nonusers in a cohort of individuals without liver disease at baseline.^16^ Our finding may be due to confounding by indication—as patients who are worked up for transplant may have more risk factors discovered and treated than those not.

We found that Hispanic/Latino ethnicity compared to White had higher associations with receiving a liver transplantation. This may be due to the referral patterns at UCSF whereas many patients are primarily managed by local physicians up until the point of advanced liver disease requiring potential transplantation, at which point they are referred to UCSF for an expedited evaluation. This signal of association would not be entirely controlled for by the other features in the Cox model, and would more likely manifest in the significant coefficient for Hispanic/Latino ethnicity. Other potential explanations for this finding include other socioeconomic, genetic or behavioral factors associated with this ethnicity that may accelerate disease progression.

There are several limitations to this study. First, the quality of the underlying data used for all modeling activities reflect actual clinical practice and may not be as robust as those captured in prospective registries. These considerations include potential measurement bias, such as miscoding, and missing data due to patient-related factors (noncompliance), physician factors (practice style), data migration errors, out of system covariates and events (ie, variceal hemorrhage). Second, our detection of MASH patients using NLP is imperfect relative to manual review, in part due to the improper redaction of protected health information from notes. We considered hepatology or gastroenterology clinical notes as the gold standard for the ascertainment of MASH within patients. We did not use noninvasive tests, as their use has evolved over the past decades. Moreover, the NASHdetection algorithm was validated by reviewing notes randomly selected throughout the entire inclusion window of 10 years to ensure that changes in the perception of the disease were still accurately captured over time. This approach allows us to keep our inclusion criteria consistent throughout the time period and avoid selection bias. Third, other variables of interest are not easily abstractable from notes—for instance, alcohol consumption is currently being captured in free text rather than as structured data. This allows for the possibility of coliver disease among patients, including those who have been diagnosed with MASH by expert clinicians, as documented in clinical notes. Lastly, although the source cohort is drawn from an urban medical center with a diverse patient population and a large catchment zone, UCSF is a tertiary care health system and is enriched for patients at a greater baseline risk of major clinical events compared to other cohorts. This study did not have access to the necessary covariates to optimally model the role of these potential selection biases on our outcomes.

## Conclusion

This study defined and characterized a diverse cohort of patients with MASH who received the standard of care at UCSF over a 10-year period. This cohort had a significant but expected burden of metabolic risk factors (HTN, T2DM, obesity, and HLD) and related comorbidities at baseline. We also developed and validated a NLP-based method for accurately identifying these patients from EHR data and identified potential predictors of progression of MASH disease. These findings lay the groundwork for future studies of disease subgroups, such as those with baseline cirrhosis, and can be used to develop future decision support tools to improve the timeliness of specialist referrals. These findings can also help support the design of future interventional trials designed to treat MASH patients, reduce their risks of serious outcomes, and curb the significant healthcare resource utilization associated with this disease.

## References

[1] Younossi Z., Anstee Q.M., Marietti M. (2018). Global burden of NAFLD and NASH: trends, predictions, risk factors and prevention. Nat Rev Gastroenterol Hepatol.

[2] Pal P., Palui R., Ray S. (2021). Heterogeneity of non-alcoholic fatty liver disease: implications for clinical practice and research activity. World J Hepatol.

[3] Kardashian A., Wilder J., Terrault N.A. (2021). Addressing social determinants of liver disease during the COVID-19 pandemic and beyond: a call to action. Hepatology.

[4] Ciardullo S., Perseghin G. (2021). Prevalence of NAFLD, MAFLD and associated advanced fibrosis in the contemporary United States population. Liver Int.

[5] Sterling R.K., Duarte-Rojo A., Patel K. (2024). AASLD practice guideline on imaging-based non-invasive liver disease assessments of hepatic fibrosis and steatosis. Hepatology.

[6] Corey K.E., Kartoun U., Zheng H. (2016). Development and validation of an algorithm to identify nonalcoholic fatty liver disease in the electronic medical record. Dig Dis Sci.

[7] Van Vleck T.T., Chan L., Coca S.G. (2019). Augmented intelligence with natural language processing applied to electronic health records for identifying patients with non-alcoholic fatty liver disease at risk for disease progression. Int J Med Inform.

[8] Czarnecka K., Czarnecka P., Tronina O. (2024). MASH continues as a significant burden on metabolic health of liver recipients. Transpl Proc.

[9] Stepanova M., Rafiq N., Makhlouf H. (2013). Predictors of all-cause mortality and liver-related mortality in patients with non-alcoholic fatty liver disease (NAFLD). Dig Dis Sci.

[10] Hamid O., Eltelbany A., Mohammed A. (2022). The epidemiology of non-alcoholic steatohepatitis (NASH) in the United States between 2010-2020: a population-based study. Ann Hepatol.

[11] Charlson M.E., Pompei P., Ales K.L. (1987). A new method of classifying prognostic comorbidity in longitudinal studies: development and validation. J Chronic Dis.

[12] Lin Y., Gong X., Li X. (2020). Distinct cause of death profiles of hospitalized non-alcoholic fatty liver disease: a 10 years' cross-sectional multicenter study in China. Front Med (Lausanne).

[13] Kanwar P., Kowdley K.V. (2016). The metabolic syndrome and its influence on nonalcoholic steatohepatitis. Clin Liver Dis.

[14] Kim W.R., Mannalithara A., Heimbach J.K. (2021). Meld 3.0: the model for end-stage liver disease updated for the modern era. Gastroenterology.

[15] Berzigotti A., Seijo S., Arena U. (2013). Elastography, spleen size, and platelet count identify portal hypertension in patients with compensated cirrhosis. Gastroenterology.

[16] Vell M.S., Loomba R., Krishnan A. (2023). Association of statin use with risk of liver disease, hepatocellular carcinoma, and liver-related mortality. JAMA Netw Open.

