# Supplementary material for: Risk Prediction in Patients With Metabolic Dysfunction–Associated Steatohepatitis Using Natural Language Processing
Source: Gastro Hep Adv. 2025 May 14;4(9):100701. doi: 10.1016/j.gastha.2025.100701 (PMC12271788; doi:10.1016/j.gastha.2025.100701)
Supplement: Supplementary Material [file mmc1.docx]

MASH Manuscript Supplemental tables and figures

**NASHDetection Methods**

We retrieved all notes associated with a Hepatology or Gastroenterology encounter at the University of California, San Francisco between 2012-2022. Notes were filtered to include only relevant note types (Supplementary Table 1). Notes written by non-Physician providers were excluded. Notes were further filtered using regular expressions to identify notes which contain an ‘ASSESSMENT’, ‘Assessment:’, ‘Assessment and Plan’, ‘Assessment/Plan’ or ‘Assessment and Recommendation’ section.

The NASHDetection algorithm was created by combining a set of MASH-related keywords to be included with a set of negation phrases relating to NASH to be removed. Regular expressions were used to replace keywords within this set of negation phrases with a generic ‘##negatedtermremoved##’ marker. The set of negation phrases were obtained first by liaising with an attending Hepatologist to manually curate common phrases containing the terms ‘NASH’/’MASH’ or ‘non-alcoholic steatohepatitis’/’metabolic syndrome-associated steatohepatitis’ but which might generate false positive results (e.g ‘low suspicion for NASH’). After replacing these terms with the generic marker in the main training corpus, we then inspected the corpus for commonly repeated phrases containing ‘NASH’. We retrieved the 50 characters up- and downstream of subsequent mentions of ‘NASH’/’MASH’ or ‘non-alcoholic steatohepatitis’/’metabolic-syndrome associated steatohepatitis’ and inspected the most common occurrences of these phrases to curate additional lists of false positive keywords and/or phrases to remove (Supplementary Table 2). This process was repeated and a final list of keywords and/or phrases was confirmed after several rounds of iteration.

We created separate development and test sets by combining two sampling approaches. In the first sample, we selected 100 notes randomly sampled from the corpus of Hepatology/Gastroenterology notes which contain an ‘Assessment’ section. This sample allows an evaluation of the use of our keywords (‘NASH’/’MASH’ or ‘non-alcoholic steatohepatitis’/’metabolic syndrome associated steatohepatitis’) in the NASHDetection pipeline. In the second sample, we selected 200 notes randomly sampled from the corpus of Hepatology/Gastroenterology notes containing both an ‘Assessment’ section and any of the keywords included in the NASHDetection pipeline (‘NASH’, ‘non-alcoholic steatohepatitis’, ‘nonalcoholic steatohepatitis’, non alcoholic steatohepatitis’, ‘MASH’, ‘metabolic syndrome associated-steatohepatitis’, ‘metabolic syndrome associated steatohepatitis’). This sample allows an evaluation of NASHDetection’s performance in negation detection, where false-positive mentions of the keywords that do not represent a formal diagnosis of NASH/MASH are evaluated. These two samples were combined, with half of each sample assigned to the development set and half to the held-out test set.

We tested NASHDetection performance on the development set of 150 notes. Scores for each of the two samples which made up the development set were evaluated and the NASHDetection algorithm was finalized. After finalizing the NASHDetection algorithm, we tested performance on the previously unseen test set of 150 notes.

**Supplementary Table 1.** Counts of included note types associated with a Hepatology or Gastroenterology encounter at UCSF between 2012-2022.

| **Note type** | **Count** |
| --- | --- |
| Progress Notes | 134205 |
| Letter | 64675 |
| Telephone Encounter | 63858 |
| Letters associated with encounter | 22355 |
| Addendum Note | 8322 |
| Assessment & Plan Note | 3149 |
| ECONSULT | 1686 |
| GI | 653 |
| H&P | 220 |
| Consults | 8 |
| Consult | 4 |

**Supplementary Table 2**. NASHDetection keywords: inclusion and exclusion criteria.

Bidirectional stepwise elimination modeling methods

|  |  |
| --- | --- |
| Keywords included | 'nonalcoholic steatohepatitis', 'non-alcoholic steatohepatitis', 'non alcoholic steatohepatitis', 'NASH', 'MASH', 'metabolic syndrome-associated steatohepatitis', 'metabolic syndrome associated steatohepatitis' |
| Keywords excluded (NASH-related): | 'without NASH', 'not consistent with NASH', 'low suspicion for NASH', 'no evidence of NASH', 'lack of NASH risk factors', 'no NASH', 'may be NASH', 'not mention NASH', 'no evidence of nonalcoholic steatohepatitis', 'no evidence of non-alcoholic steatohepatitis', 'no evidence of non alcoholic steatohepatitis', 'without NASH' , 'NOT consistent with NASH' , 'low suspicion for NASH', 'no evidence of NASH' , 'lack of NASH risk factors' , 'no NASH' , 'less likely for her to have recurrent NASH' , 'did not have evidence of NASH' , 'does have risk factors for NAFLD/NASH' , 'without evidence of steatohepatitis' , 'not meeting criteria for diagnosis of NASH' , 'not classical for NASH' , 'presence of NASH is unlikely' , 'No clear NASH' , 'not have active NASH' , 'no evidence of active steatohepatitis' , 'did not have significant fibrosis or evidence of inflammatory ***** concerning for NASH' , 'no evidence of steatohepatitis', 'shares many pathogenic features in common with NASH' , 'determine if he has NASH' , 'monitor for nonalcoholic steatohepatitis', 'monitor for non-alcoholic steatohepatitis', 'monitor for non alcoholic steatohepatitis' , 'evaluate for possible NASH' , 'Phase III study will be started soon in patients with NASH' , 'We did not spend much time discussing ***** vs. NASH and will discuss this more at our next visit if the diagnosis is clear.' , 'would consider pursuing liver biopsy to assess for AIH vs NASH' , 'risk for progression to NASH' , 'consideration for liver biopsy to assess for etiology of elevated liver enzymes such as seronegative AIH, NASH' , 'assess for NASH' , 'prevent development of concurrent NASH' , 'concerning for underlying inflammation and NASH' , 'high risk for progression to NASH' , 'initially thought to relate to alcohol or combination of ***** and NASH' , 'Patient had NASH fibrosis score checked' , 'liver tests would be atypical for NAFLD/NASH alone' , 'vs NASH' , 'assess for nonalcoholic steatohepatitis (NASH)' , 'assess for non alcoholic steatohepatitis (NASH)' , 'assess for non-alcoholic steatohepatitis (NASH)' , 'association between NASH and PCOS' , 'has been associated with development of fatty liver and steatohepatitis' , 'evaluate for AIH +/- NASH' , 'at risk for concurrent NAFLD and NASH' , 'risk factors for NASH' , 'also seen with NASH' , 'NASH risk factors' , 'assessment of NASH' , 'risk for non-alcoholic steatohepatitis' , 'risk for nonalcoholic steatohepatitis' , 'risk for non alcoholic steatohepatitis' , 'risk factors for the development of NASH' , 'may relate to NAFLD/NASH' , 'assess for NASH' , 'rule out NASH' , 'potential for progression to NASH' , 'Vitamin E is reserved for biopsy-proven NASH' , 'risk for developing NAFLD/NASH' , 'risk for NASH' , 'clarify whether this presents NASH' , 'evaluate for NASH' , 'evaluate the presence of NASH' , 'differential diagnosis for cirrhosis in this patient includes alcoholic cirrhosis, NASH', 'Liver biopsy is required to distinguish between so-called '*****' steatosis (no inflammation) and steatohepatitis (NASH)', 'to differentiate between benign steatosis vs. nonalcoholic steatohepatitis is to do a liver biopsy', '-- NASH: A1c, Lipid panel', 'We did not spend much time discussing ***** vs. NASH and will discuss this more at our next visit', 'without inflammation, steatohepatitis (NASH; fat + inflammation in a characteristic pattern)', 'Vitamin E has been demonstrated to improve hepatic steatosis and inflammation in non-diabetic patients with NASH.', 'steatosis (no inflammation) and steatohepatitis (NASH)', 'Only patients with biopsy-proven NASH should be treated with vitamin E.', 'whereas NASH can progress to cirrhosis.', 'inflammation in non-diabetic patients with NASH.', 'low risk category for NASH', ' or NASH', ' or non-alcoholic steatohepatitis', ' or nonalcoholic steatohepatitis', ' or non alcoholic steatohepatitis', 'vs. NASH', 'vs NASH', 'vs. non-alcoholic steatohepatitis', 'vs non-alcoholic steatohepatitis', 'vs. non alcoholic steatohepatitis', 'vs non alcoholic steatohepatitis', 'vs. nonalcoholic steatohepatitis', 'vs nonalcoholic steatohepatitis', 'If found to have NAFLD we will discuss the role of liver biopsy which is currently required for the diagnosis of fat related inflammation and scarring known as non alcoholic steatohepatitis (NASH)', 'between ***** and NASH', 'did not have clear NASH', 'The use of vitamin E should be reserved only for patients with biopsy-proven NASH', 'NAFLD is an umbrella term that encompasses both nonalcoholic fatty liver (*****) or non-alcoholic steatohepatitis (NASH).', 'no risk factors for nonalcoholic steatohepatitis', 'vitamin E is reserved for patients with biopsy proven NASH', 'the typical causes of abnormal liver function tests lasting for longer than 6 months: NAFLD/NASH', 'the typical causes of abnormal liver function tests lasting for longer than 6 months: NAFLD/NASH', 'Liver biopsy is required to distinguish between ... Only patients with biopsy-proven', 'Liver biopsy is required to distinguish between ... patients with biopsy-proven NASH' |
| Keywords excluded (MASH-related): | 'without MASH', 'not consistent with MASH', 'low suspicion for MASH', 'no evidence of MASH', 'lack of MASH risk factors', 'no MASH', 'may be MASH', 'not mention MASH', 'no evidence of metabolic dysfunction-associated steatohepatitis', 'no evidence of metabolic dysfunction associated steatohepatitis', 'without MASH' , 'NOT consistent with MASH' , 'low suspicion for MASH', 'no evidence of MASH' , 'lack of MASH risk factors' , 'no MASH' , 'less likely for her to have recurrent MASH' , 'did not have evidence of MASH' , 'does have risk factors for MAFLD/MASH' , 'without evidence of steatohepatitis' , 'not meeting criteria for diagnosis of MASH' , 'not classical for MASH' , 'presence of MASH is unlikely' , 'No clear MASH' , 'not have active MASH' , 'no evidence of active steatohepatitis' , 'did not have significant fibrosis or evidence of inflammatory ***** concerning for MASH' , 'no evidence of steatohepatitis', 'shares many pathogenic features in common with MASH' , 'determine if he has MASH' , 'monitor for metabolic dysfunction associated steatohepatitis' ,'monitor for metabolic dysfunction-associated steatohepatitis' , 'evaluate for possible MASH' , 'Phase III study will be started soon in patients with MASH' , 'We did not spend much time discussing ***** vs. MASH and will discuss this more at our next visit if the diagnosis is clear.' , 'would consider pursuing liver biopsy to assess for AIH vs MASH' , 'risk for progression to MASH' , 'consideration for liver biopsy to assess for etiology of elevated liver enzymes such as seronegative AIH, MASH' , 'assess for MASH' , 'prevent development of concurrent MASH' , 'concerning for underlying inflammation and MASH' , 'high risk for progression to MASH' , 'initially thought to relate to alcohol or combination of ***** and MASH' , 'Patient had MASH fibrosis score checked' , 'liver tests would be atypical for MAFLD/MASH alone' , 'vs MASH' , 'assess for metabolic dysfunction associated steatohepatitis (MASH)' , 'assess for metabolic dysfunction-associated steatohepatitis (MASH)' , 'association between MASH and PCOS' , 'has been associated with development of fatty liver and steatohepatitis' , 'evaluate for AIH +/- MASH' , 'at risk for concurrent MAFLD and MASH' , 'risk factors for MASH' , 'also seen with MASH' , 'MASH risk factors' , 'assessment of MASH' , 'risk for metabolic dysfunction associated steatohepatitis' , 'risk for metabolic dysfunction-associated steatohepatitis' , 'risk factors for the development of MASH' , 'may relate to MAFLD/MASH' , 'assess for MASH' , 'rule out MASH' , 'potential for progression to MASH' , 'Vitamin E is reserved for biopsy-proven MASH' , 'risk for developing MAFLD/MASH' , 'risk for MASH' , 'clarify whether this presents MASH' , 'evaluate for MASH' , 'evaluate the presence of MASH' , 'differential diagnosis for cirrhosis in this patient includes alcoholic cirrhosis, MASH', 'Liver biopsy is required to distinguish between so-called '*****' steatosis (no inflammation) and steatohepatitis (MASH)', 'to differentiate between benign steatosis vs. metabolic dysfunction-associated steatohepatitis is to do a liver biopsy', '-- MASH: A1c, Lipid panel', 'We did not spend much time discussing ***** vs. MASH and will discuss this more at our next visit', 'without inflammation, steatohepatitis (MASH; fat + inflammation in a characteristic pattern)', 'Vitamin E has been demonstrated to improve hepatic steatosis and inflammation in non-diabetic patients with MASH.', 'steatosis (no inflammation) and steatohepatitis (MASH)', 'Only patients with biopsy-proven MASH should be treated with vitamin E.', 'whereas MASH can progress to cirrhosis.', 'inflammation in non-diabetic patients with MASH.', 'low risk category for MASH', ' or MASH', ' or metabolic dysfunction associated steatohepatitis', ' or metabolic dysfunction-associated steatohepatitis', 'vs. MASH', 'vs MASH', 'vs. metabolic dysfunction associated steatohepatitis', 'vs metabolic dysfunction-associated steatohepatitis', 'If found to have MAFLD we will discuss the role of liver biopsy which is currently required for the diagnosis of fat related inflammation and scarring known as metabolic dysfunction-associated steatohepatitis (MASH)', 'between ***** and MASH', 'did not have clear MASH', 'The use of vitamin E should be reserved only for patients with biopsy-proven MASH', 'MAFLD is an umbrella term that encompasses both metabolic dysfunction-associated fatty liver (*****) or metabolic dysfunction-associated steatohepatitis (MASH).', 'no risk factors for metabolic dysfunction-associated steatohepatitis', 'vitamin E is reserved for patients with biopsy proven MASH', 'the typical causes of abnormal liver function tests lasting for longer than 6 months: MAFLD/MASH', 'Liver biopsy is required to distinguish between ... patients with biopsy-proven MASH' |

**Supplementary Figure 1: Study Design.** Participants were included based on records available between 2012 and 2022. Follow-up for primary and secondary outcomes was conducted from the index date through December 31, 2023. ICD: International classification of disease, EHR: Electronic Health Records


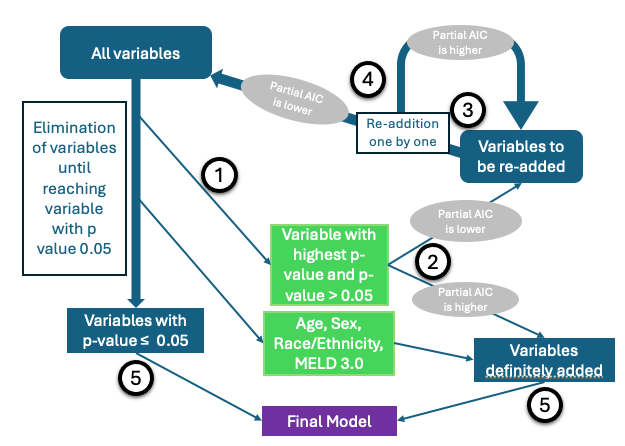


**Supplementary Figure 2: Bidirectional stepwise variable selection procedure.** We followed the following steps: 1) Remove the variable with the highest p-value, 2) If the Akaike information criterion (AIC) decreased, the variable was removed from the model; if the AIC increased, the variable was added into the model. 3) The previously removed variables were one by one re-added in the model. 4) If the AIC increased, the variable was removed again, if the AIC decreased the variable will be readded in the model, 5). Repeat steps 1, 2 and 3, 4 until reaching variables with a p-value ≤ 0.05

The following variables were kept in all models and not subject to variable selection: Age, Sex, Race/Ethnicity and calculated baseline MELD 3.0 scores. This was done because of their a priori importance, and to help stabilize the estimates of other variables.

**Supplemental table 3**: Baseline covariates selected a priori for time to event analysis. (Supplemental methods).

| Characteristics | Age at time of MASH diagnosis, MASH diagnosis in pre/post COVID era, sex, insurance (Medicare, Medicaid, commercial, and self-pay/other), area deprivation index, residential driving distance to UCSF, Race (White, Asian, African American, Native American/Alaskan/Hawaiian/Other, and unknown/declined), and Hispanic/Latino ethnicity | UCSF structured data: Variables |
| --- | --- | --- |
| Labs / vitals | Body mass index | UCSF structured data: Variable |
|  | Systolic blood pressure | UCSF structured data: Variable |
|  | Diastolic blood pressure | UCSF structured data: Variable |
|  | alanine aminotransferase | Label lab test: 'alanine transaminase', 'alt - external', 'alt (sgpt) (hcvf):', 'alt', 'alt (sgpt) p5p' |
|  | aspartate aminotransferase | Label lab test: 'ast', 'ast - external', 'liver panel (alkp,ast)' |
|  | LDL Cholesterol | Label lab test: 'ldl cholesterol', 'cholesterol, ldl', 'ldl, small', 'ldl cholesterol - external', 'ldl pattern', 'real ldl size pattern', 'ldl direct', 'sum total ldl-c', 'ldl, large a', 'ldl particle number', 'ldlr', 'ldl, very small b', 'ldl peak size', 'total ldl-chol dir', 'ldl, large b', 'ldl, medium', 'ldl, very small c', 'ldl cholesterol -cvri', 'ldl, very small a', 'ldl, very small d' |
|  | Hemoglobin A1c | Label lab test: 'hemoglobin a1c', 'hemoglobin a1c - external', 'hemoglobin a1c (alternate method)', 'hemoglobin a1c (%nspg)' |
|  | Creatinine | Label lab test: 'creatinine, serum - external', 'creatinine', 'creatinine whole blood', 'creatinine, wbld', 'creatinine, serum / plasma', 'creatinine, serum', 'creatine, serum', 'creatinine, random u', 'poc creatinine, istat', 'creatinine, istat', 'creatinine, istat (bopc only)' |
|  | estimated glomerular filtration rate (eGFR) | Label lab test: 'egfr - high estimate', 'egfr - low estimate', 'egfr (high estimate) - external', 'egfr (low estimate) - external', 'egfrcys', 'estimated glomerular filtration rate', 'egfr mutation testing', 'egfrcr', 'creat with egfr', 'egfrcr-cys' |
|  | Albumin | Label lab test: 'albumin, serum / plasma', 'albumin, serum - external', 'albumin, spep', 'albumin conc.', 'albumin, serum', 'albumin' |
|  | alpha-fetoprotein | Label lab test: 'afp, serum, tumor marker', 'alpha-fetoprotein, serum', 'afp - external', 'alpha fetoprotein, total', 'alpha fetoprotein, l3 pct', 'afp - l3% - external', 'afp-l3%', 'california afp screening', 'afp, serum, tumor marker (external lab)' |
|  | Platelet count | Label lab test: 'platelets - external', 'platelets - units ready', 'platelet count', 'platelet estimate', 'platelet crossmatch', 'platelet count phase', 'platelet aggregation', 'heparin induced platelet antibody', 'platelet ab screen, flow cytometry', 'platelets', 'cbc w/platelet count', 'check plt smr vs cnt', 'plt agg interp (md):', 'citrated platelet count', 'platelet count (sodium citrate)', 'plt assoc ig, dir' |
|  | alkaline phosphatase | Label lab test: 'alkaline phosphatase', 'alkaline phosphatase - external', 'liver panel (alkp,ast)' |
|  | prothrombin time/INR | Label lab test: 'inr - external', 'inr', 'inr - acelis home meter', 'dilute prothrombin time(dpt)' |
|  | Bilirubin | Label lab test: 'bilirubin, total', 'bilirubin, direct', 'bilirubin, total - external', 'bilirubin, direct - external', 'bilirubin, indirect', 'bilirubin,total (02)', 'total bilirubin', 'bilirubin', 'bilirubin, indirect - external', 'bilirubin direct', 'bilirubin total/direct - external', 'bilirubin, total (external lab)' |
|  | Triglycerides | Label lab test: 'triglycerides, serum', 'triglycerides - external', 'triglycerides-direct' |
|  | Sodium | 'sodium, serum / plasma', 'sodium, whole blood', 'sodium - external', 'sodium, wbld' |
|  | MELD 3.0 score | Extracted from Label lab test: 'meld match score - external', 'meld/peld - external', 'meld'  Or calculated from formula:  Female: 1.33 + 4.56 * np.log(row['value_bili']) + 0.82 * (137 - row['value_na']) - 0.24 * (137 - row['value_na']) * np.log(row['value_bili']) + 9.09 * np.log(row['value_pt']) + 11.14 * np.log(row['value_creat']) + 1.85 * (3.5 - row['value_alb']) - 1.83 * (3.5 - row['value_alb']) * np.log(row['value_creat']) + 6  Male: 4.56 * np.log(row['value_bili']) + 0.82 * (137 - row['value_na']) - 0.24 * (137 - row['value_na']) * np.log(row['value_bili']) + 9.09 * np.log(row['value_pt']) + 11.14 * np.log(row['value_creat']) + 1.85 * (3.5 - row['value_alb']) - 1.83 * (3.5 - row['value_alb']) * np.log(row['value_creat']) + 6 |
| Medications |  | Metformin, insulin, ≥ 2 anti-HTN medications, aspirin, any  thiazolidinedione, any statin, any sulfonylurea, any SGLT-2 inhibitor, any GLP-1 receptor agonist, any DPP-4 inhibitor, any ACE and/or ARB |
|  | Aspirin | Labels include [‘aspirin’, ‘acetylsalicylic’] AND NOT [‘amiprin’, ‘aspirin-free’, ‘aspirin free’, 'dihydrocodeine comp mod capsule', 'dihydroxcodeine compound capsule', 'menstrual 500 mg', 'midol', 'n/aspirin', 'no aspirin', 'no-aspirin', 'non aspirin', 'non-aspirin', 'non-acetylsalicylic', 'w/o aspirin', 'without aspirin', 'sinodeine capsule'] |
|  | Insulin | Labels include ['insulin regular','admelog','afrezza','apidra','basaglar','novolog','exubera','fiasp','humalog','humulin','iletin','insulatard','insulin','insulin aspar','insulin aspart','insulin beef','degludec','detemir','glargine','glargine-aglr','glargine-yfgn','glulisine','insulin human','insulin inhalation','insulin isophane','insulin l','insulin lente','lispro','lispro-aabc','insulin nph','insulin nph-reg','insulin nph-regular','insulin pork','insulin protamine','insulin pump','insulin purified','insulin r','insulin u-100','insulin zinc','inv insulin','lantus','lentard','levemir','lyumjev','mixtard','monotard','myxredlin','novolin','relion n','relion r','rezvoglar','semglee','soliqua','toujeo','tresiba','velosulin','xultophy'] |
|  | Statin | Labels include ['advicor’, ‘altocor’, ‘altoprev’, ‘atorvaliq’, ‘atorvastatin’, ‘baycol’, ‘caduet’, ‘cerivastatin’, ‘crestor’, ‘ezallor’, ‘flolipid’, ‘fluvastatin’, ‘juvisync’, ‘lescol’, ‘lipitor’, ‘liptruzet’, ‘livalo’, ‘lovastatin’, ‘mevacor’, ‘pitavastatin’, ‘pravachol’, ‘pravastatin’, ‘pravigard’, ‘rosuvastatin’, ‘roszet’, ‘simcor’, ‘simvastatin’, ‘vytorin’, ‘zocor’, ‘zypitamag'] AND NOT ['nystat’, ‘placebo'] |
|  | Metformin | Labels include ['actoplus’, ‘appformin’, ‘avandamet’, ‘fortamet’, ‘glucophage’, ‘glumetza’, ‘invokamet’, ‘janumet’, ‘jentadueto’, ‘kazano’, ‘kombiglyze’, ‘metaglip’, ‘metformin’, ‘riomet’, ‘segluromet’, ‘synjardy’, ‘trijardy’, ‘xigduo'] |
|  | Thiazolidinedione | Labels include ['actoplus’, ‘actos’, ‘avandamet’, ‘avandaryl’, ‘avandia’, ‘duetact’, ‘oseni’, ‘pioglitazone’, ‘rezulin’, ‘rosiglitazone’, ‘troglitazone' ] AND NOT [‘alpha-d-galactosidase’, ‘baby’, ‘beano’, ‘boost’, ‘bacid’, ‘bactoshield’, ‘bright beginnings’, ‘cebo’, ‘comple’, ‘diabetaid’, ‘digestive’, ‘ensure’, ‘food’, ‘galaxtra’, ‘gas’, ‘glucerna’, ‘gluco burst’, ‘icn-tolam’, ‘impact’, ‘isocal’, ‘isosource’, ‘isotein’, ‘jevity’, ‘kindercal’, ‘kindersprout’, ‘lactos’, ‘l-emental’, ‘nutr’, ‘oral liquid’, ‘oral packet’, ‘orange’, ‘oribetic’, ‘pedia’, ‘placebo’, ‘portagen’, ‘tolerex’, ‘vactosertib'] |
|  | sulfonylurea | Labels include ['actos’, actoplus’, acetohexamide’, amaryl’, avandaryl’, avandamet’, avandia’, chlorpropamide’, diabeta’, diabinese’, dibatrol’, duetact’, dymelor’, glimepiride’, glipizide’, glucamide’, glucotrol’, glucovance’, glycron’, glyburide’, glynase’, insulase’, metaglip’, micronase’, oseni’, pioglitazone’, ronase’, rosiglitazone’, tolazamide’, tolbutamide’, tolinase'] AND NOT ['alpha-d-galactosidase’, baby’, beano’, boost’, bacid’, bactoshield’, bright beginnings’, cebo’, comple’, diabetaid’, digestive’, ensure’, food’, galaxtra’, gas’, glucerna’, gluco burst’, icn-tolam’, impact’, isocal’, isosource’, isotein’, jevity’, kindercal’, kindersprout’, lactos’, l-emental’, nutr’, oral liquid’, oral packet’, orange’, oribetic’, pedia’, placebo’, portagen’, tolerex’, vactosertib'] |
|  | GLP-1 receptor agonist | Labels include ['adlyxin’, albiglutide’, bydureon’, byetta’, dulaglutide’, exenatide’, lixisenatide’, liraglutide’, mounjaro’, ozempic’, rybelsus’, saxenda’, semaglutide’, soliqua’, tanzeum’, tirzepatide’, trulicity’, wegovy’, xultophy'] |
|  | DPP-4 inhibitor | Labels include ['alogliptin’, saxagliptin’, glyxambi’, janumetjanuvia’, jentadueto’, juvisync’, kazano’, kombiglyze’, linagliptin’, nesina’, onglyza’, oseni’, qtern’, saxagliptin’, sitagliptin’, steglujan’, tradjenta’, trijardy'] |
|  | SGLT-2 inhibitor | Labels include ['bexagliflozin’, brenzavvy’, canagliflozin’, dapagliflozin’, empagliflozin’, ertugliflozin’, farxiga’, glyxambi’, inpefa’, invokamet’, invokana’, jardiance’, qtern’, segluromet’, sotagliflozin’, steglatro’, steglujan’, synjardy’, trijardy’, xigduo'] |
|  | ACE inhibitor | Labels include ['accupril’, aceon’, altace’, benazepril’, capoten’, captopril’, enalapril’, enalaprilat’, epaned’, fosinopril’, lisinopril’, lotensin’, mavik’, moexipril’, monopril’, perindopril’, prinivil’, qbrelis’, quinapril’, ramipril’, trandolapril’, univasc’, vasotec’, zestril'] AND NOT ['placebo'] |
|  | ARB (ARAII) | Labels include ['atacand’, avapro’, avalide’, azilsartan’, azor’, benicar’, candesartan’, cozaar’, diovan’, edarbi’, eprosartan’, irbesartan’, losartan’, micardis’, olmesartan’, telmisartan’, teveten’, valsartan'] AND NOT ['placebo'] |
|  | Other antihypertensive: Centrally acting alpha-agonist hypotensive agents | Labels include ['clonidine’, guanabenz’, guanethidine’, guanfacine’, methyldopa’, rilmenidine’, moxonidine’, tizanidine’, aldomet’, catapres’, clorpres’, combipres’, enduronyl’, esimil’, hylorel’, ingadine’, ismelin’, l-dopres’, methyclodine’, nexiclon’, tenex’, wytensin'] AND NOT ['placebo'] |
|  | Other antihypertensive: Alpha-blockers | Labels include ['doxazosin’, prazosin’, terazosin’, aquapres’, bart-serp’, cherapas’, chloroserp’, chloroserpine’, deserpidine’, diurigen’, diupres’, genutensin’, hydrap-es’, hydro-fluserpine’, hydroplus’, hydropres’, hydroprin’, hydroserpalan’, hydroserpazine’, hydroserpine’, hydrosine’, hydrotensin’, hyserp’, mallopress’, marpres’, meserpidine’, methyclodine’, oreticyl’, panpres’, q-pres’, rauwolfia’, rauzide’, renese-r’, reserpoid’, reserpine’, rondameth’, salazide’, salutensin’, sandril’, ser-a-gen’, seragen’, seralazide’, serapine’, serathide’, serpalan’, serpanray’, serpasil’, serpatab’, serpate’, serpazide’, serpex’, sertina’, sterapres’, thiaserp’, uni-serp’, unipres'] AND NOT ['placebo'] |
|  | Other antihypertensive: Beta-blockers | Label include [‘labetalol] AND NOT ['placebo'] |
|  | Other antihypertensive: Calcium Channel Blockers (CCBs) | Labels include ['amlodipine’, nifedipine’, verapamil’, diltiazem'] AND NOT ['placebo'] |
|  | Other antihypertensive: Diuretics | Labels include ['bendroflumethiazide’, chlorothiazide’, hydrochlorothiazide’, hydroflumethiazide’, chlorthalidone’, indapamide’, metolazone’, alduril’, aldoril’, apresazide’, apresodex’, aquapres’, bart-serp’, cherapas’, chloroserp’, chloroserpine’, clorpres’, combipres’, diupres’, diurigen’, enduronyl’, esimil’, flumezide’, genutensin’, hydra-zide’, hydrazide’, hydro-fluserpine’, hydroplus’, hydropres’, hydroprin’, hydroserp’, hydroserpalan’, hydroserpazine’, hydroserpine’, hydrosine’, hydrotensin’, l-dopres’, mallopress’, marpres’, methyclothiazide’, meserpidine’, methyclodine’, oreticyl’, panpres’, q-pres’, renese-r’, rondameth’, salazide’, salutensin’, ser-a-gen’, seragen’, seralazide’, serapine’, serathide’, serpazide’, serpex’, sterapres’, thiaserp’, uni-serp’, unipres'] AND NOT ['placebo'] |
|  | Other antihypertensive: Direct Renin Inhibitors | Labels include ['aliskiren'] AND NOT ['placebo'] |
|  | Other antihypertensive: Vasodilators | Labels include [‘inversine’, mecamylamine’, vecamyl'] AND NOT 'placebo' |
| Comorbidities |  | , chronic kidney disease (using eGFR), Charlson Comorbidity Index, |
|  | Cirrhosis | ICD-9 codes: '571.2', '571.5', '571.6', '572.2', '572.20', '789.5', '789.59', '456.0', '456.20', '456.1', '456.21', '573.5', '572.4'  ICD-10 codes: 'K70.30', 'K74.69', 'K74.60', 'E83.11', 'K71.7', 'K72.1', 'K74.3', 'K74.4', 'K74.5', 'K72.91', 'G93.40', 'K72.11', 'K70.41', 'K71.11', 'K72.01', 'B19.0', 'B19.11', 'B19.21', 'K70.31', 'K70.11', 'K71.51', 'R18.8', 'I85.00', 'I86.4', 'I85.1', 'I85.01', 'I86.41', 'I85.11', 'K76.81', 'K76.7' |
|  | Liver Transplant | Procedure code: '47135' |
|  | Type 2 diabetes mellitus | ICD-9: '250.00', '250.02', '250.10', '250.12', '250.20', '250.22', '250.30', '250.32', '250.40', '250.42', '250.50', '250.52', '250.60', '250.62', '250.70', '250.72', '250.80', '250.82', '250.90', '250.92'  ICD-10: 'E11.9', 'E11.8', 'E11.2', 'E11.3', 'E11.4', 'E11.5', 'E11.62', 'E11.64', 'E11.65' |
|  | Hypertension | ICD-9 codes: '401.0', '401.1', '401.2', '401.3', '401.4',' 401.5', '401.6', '401.7', '401.8', '401.9'  ICD-10 codes: 'I10', 'I27.0', 'K76.6', 'G93.2', 'I15.1', 'I15.9', 'I15.8' |
|  | Peripheral artery disease | ICD-10: 'I70.20', 'I70.21', 'I70.22', 'I70.23', 'I70.24', 'I70.25', 'I70.26', 'I70.29', 'I70.30', 'I70.31', 'I70.32', 'I70.33', 'I70.34', 'I70.35', 'I70.36', 'I70.39', 'I70.40', 'I70.41', 'I70.42', 'I70.43', 'I70.44', 'I70.45', 'I70.46', 'I70.49', 'I70.50', 'I70.51', 'I70.52', 'I70.53', 'I70.54', 'I70.55', 'I70.56', 'I70.59', 'I70.60', 'I70.61', 'I70.62', 'I70.63', 'I70.64', 'I70.65', 'I70.66', 'I70.69', 'I70.70', 'I70.71', 'I70.72', 'I70.73', 'I70.74', 'I70.75', 'I70.76', 'I70.79', 'I70.8', 'I70.92', 'I73.9'  Procedure: '39.50','39.90','38.18','84.10','84.11','84.12','84.13','84.14','84.15','84.16','84.17','84.18','84.19' |
|  | Coronary artery disease | ICD-9 codes: '410', '410.0', '410.00', '410.01', '410.02', '410.1', '410.10', '410.11', '410.12', '410.2', '410.20', '410.21', '410.22', '410.3', '410.30', '410.31', '410.32', '410.4', '410.40', '410.41', '410.42', '410.5', '410.50', '410.51', '410.52', '410.6', '410.60', '410.61', '410.62', '410.7', '410.70', '410.71', '410.72', '410.8', '410.80', '410.81', '410.82', '410.9', '410.90', '410.91', '410.92', '411', '411.0', '411.1', '411.8', '411.81', '411.89', '412', '413', '413.0', '413.1', '413.9', '414', '414.0', '414.00', '414.01', '414.02', '414.03', '414.04', '414.05', '414.06', '414.07', '414.2', '414.3', '414.4', '414.8', '414.9', '429.79', '429.71'  ICD-10 codes: 'I20', 'I20.0', 'I20.1', 'I20.1', 'I20.2', 'I20.8', 'I20.81', 'I20.89', 'I20.9', 'I21', 'I21.0', 'I21.01', 'I21.02', 'I21.09', 'I21.1', 'I21.11', 'I21.19', 'I21.2', 'I21.21', 'I21.29', 'I21.3', 'I21.4', 'I21.9', 'I21.A', 'I21.A1', 'I21.A9', 'I21.B', 'I22', 'I22.0', 'I22.1', 'I22.2', 'I22.8', 'I22.9', 'I23', 'I23.0', 'I23.1', 'I23.2', 'I23.3', 'I23.4', 'I23.5', 'I23.6', 'I23.7', 'I23.8', 'I24', 'I24.0', 'I24.1', 'I24.8', 'I24.81', 'I24.89', 'I24.9', 'I25', 'I25.1', 'I25.10', 'I25.11', 'I25.110', 'I25.111', 'I25.112', 'I25.118', 'I25.119', 'I25.2', 'I25.3', 'I25.4', 'I25.41', 'I25.42', 'I25.5', 'I25.6', 'I25.7', 'I25.70', 'I25.700', 'I25.701', 'I25.702', 'I25.708', 'I25.709', 'I25.71', 'I25.710', 'I25.711', 'I25.712', 'I25.718', 'I25.719', 'I25.72', 'I25.720', 'I25.721', 'I25.722', 'I25.728', 'I25.729', 'I25.73', 'I25.730', 'I25.731', 'I25.732', 'I25.738', 'I25.739', 'I25.75', 'I25.750', 'I25.751', 'I25.752', 'I25.758', 'I25.759', 'I25.76', 'I25.760', 'I25.761', 'I25.762', 'I25.768', 'I25.769', 'I25.79', 'I25.790', 'I25.791', 'I25.792', 'I25.798', 'I25.799', 'I25.8', 'I25.81', 'I25.810', 'I25.811', 'I25.812', 'I25.82', 'I25.83', 'I25.84', 'I25.85', 'I25.89', 'I25.9' |
|  | chronic kidney disease (using eGFR) | Defined from eGFR in labs |
|  | Obstructive sleep apnea | ICD-9 codes: '327.20', '327.23', '780.51', '780.53', '780.57'  ICD-10 codes: 'G4730', 'G4733', 'G4739' |
|  | History of bariatric surgery | ICD-10 codes: 'Z98.84'  Procedure code: '43644', '43645', '43770', '43845', '43846', '43847', '43775' |
|  | Hyperlipidemia | ICD-9 codes: '272.0', '272.1', '272.2', '272.3', '272.4'  ICD-10 codes: 'E78.0', 'E78.00', 'E78.01', 'E78.1', 'E78.2', 'E78.3', 'E78.4','E78.41','E78.49','E78.5' |
|  | Heart failure | ICD-9 codes: 428.0','428.1', '428.20', '428.21', '428.22', '428.23', '428.30','428.31','428.32','428.33', '428.40','428.41','428.42','428.43', '428.9'  ICD-10 codes: 'I50.1', 'I50.20', 'I50.21', 'I50.22', 'I50.23', 'I50.30', 'I50.31', 'I50.32', 'I50.33', 'I50.40', 'I50.41', 'I50.42', 'I50.43', 'I50.810', 'I50.811', 'I50.812', 'I50.813', 'I50.814', 'I50.82', 'I50.83', 'I50.84', 'I50.89', 'I50.9' |
|  | Charlson Comorbidity Index, | Myocardial infarction (1 point):  icd9 = ['410.1', '410.2', '410.3', '410.4', '410.5', '410.6', '410.7', '410.8', '410.9', '412.1', '412.2', '412.3', '412.4', '412.5', '412.6', '412.7', '412.8', '412.9'],  icd10 = ['I21.1', 'I21.2', 'I21.3', 'I21.4', 'I21.5', 'I21.6', 'I21.9', 'I22.1', 'I22.2', 'I22.3', 'I22.4', 'I22.5', 'I22.6', 'I22.8', 'I22.9', 'I25.2', 'I25.21', 'I25.22', 'I25.29']  Congestive heart failure (1 point):  icd9 = ['428.1', '428.2', '428.3', '428.4', '428.5', '428.6', '428.7', '428.8', '428.9'],  icd10 = ['I09.9', 'I11.0', 'I13.0', 'I13.2', 'I25.5', 'I42.0', 'I42.5', 'I42.6', 'I42.7', 'I42.8', 'I42.9', 'I43', 'I43.0', 'I43.1', 'I43.2', 'I43.8', 'I43.9', 'I50', 'I50.0', 'I50.1', 'I50.9', 'P29.0', '398.91', '402.01', '402.11', '402.91', '404.01', '404.03', '404.11', '404.13', '404.91', '404.93', '425.4', '425.5', '425.6', '425.7', '425.8', '425.9']  Peripheral vascular disease (1 point):  icd9 = ['441.1', '441.2', '441.3', '441.4', '441.5', '441.6', '441.7', '441.8', '441.9', '443.91', '443.92', '443.93', '443.94', '443.99', '785.4', '785.41', 'V43.4', 'V43.41']  icd10 = ['I70.0', 'I70.1', 'I70.2', 'I70.3', 'I70.4', 'I70.5', 'I70.6', 'I70.8', 'I70.9', 'I71.0', 'I71.1', 'I71.2', 'I71.3', 'I71.4', 'I71.5', 'I71.6', 'I71.8', 'I71.9', 'I73.1', 'I73.8', 'I73.9', 'I77.1', 'I79.0', 'I79.2', 'K55.1', 'K55.8', 'K55.9', 'Z95.8', 'Z95.9', '093.0', '437.3', '440.0', '440.1', '440.2', '440.3', '440.8', '440.9', '441.0', '441.1', '441.2', '441.3', '441.4', '441.5', '441.6', '441.7', '441.8', '441.9', '443.1', '443.2', '443.3', '443.8', '443.9', '447.1', '557.1', '557.9', 'V43.4']  Cerebrovascular disease (1 point):  icd9 = ['430.0', '430.1', '430.2', '430.3', '430.4', '430.5', '430.6', '430.7', '430.8', '430.9', '431.0', '431.1', '431.2', '431.3', '431.4', '431.5', '431.6', '431.7', '431.8', '431.9', '432.0', '432.1', '432.9', '433.0', '433.1', '433.2', '433.3', '433.8', '433.9', '434.0', '434.1', '434.9', '435.0', '435.1', '435.2', '435.3', '435.8', '435.9', '436.0', '436.1', '436.2', '437.0', '437.1', '437.2', '437.3', '437.4', '437.5', '437.6', '437.7', '437.8', '437.9', '438.0', '438.1', '438.2', '438.3', '438.4', '438.5', '438.6', '438.7', '438.8', '438.9'],  icd10 = ['G45.0', 'G45.1', 'G45.2', 'G45.8', 'G45.9', 'G46.0', 'G46.1', 'G46.2', 'G46.8', 'G46.9', 'H34.0', 'I60.0', 'I60.1', 'I60.2', 'I60.3', 'I60.4', 'I60.5', 'I60.6', 'I60.7', 'I60.8', 'I60.9', 'I61.0', 'I61.1', 'I61.9', 'I62.0', 'I62.1', 'I62.9', 'I63.0', 'I63.1', 'I63.2', 'I63.3', 'I63.4', 'I63.5', 'I63.6', 'I63.8', 'I63.9', 'I64', 'I65.0', 'I65.1', 'I65.2', 'I65.8', 'I65.9', 'I66.0', 'I66.1', 'I66.2', 'I66.8', 'I66.9', 'I67.0', 'I67.1', 'I67.2', 'I67.3', 'I67.4', 'I67.5', 'I67.6', 'I67.7', 'I67.8', 'I67.9', 'I68.0', 'I68.1', 'I68.2', 'I68.8', 'I68.9', 'I69.0', 'I69.1', 'I69.2', 'I69.3', 'I69.4', 'I69.8', 'I69.9', '362.34']  Dementia (1 point):  icd9 = ['290.0', '290.1', '290.2', '290.3', '290.4', '290.9']  icd10 = ['F00.0', 'F00.1', 'F00.2', 'F00.3', 'F00.9', 'F01.0', 'F01.1', 'F01.2', 'F01.3', 'F01.8', 'F01.9', 'F02.0', 'F02.1', 'F02.2', 'F02.3', 'F02.4', 'F02.8', 'F02.9', 'F03.0', 'F03.1', 'F03.2', 'F03.8', 'F03.9', 'F05.1', 'G30.0', 'G30.1', 'G30.8', 'G30.9', 'G31.1', '290.10', '290.11', '290.12', '290.13', '290.20', '290.21', '290.3', '290.40', '290.41', '290.42', '290.8', '290.9', '294.1', '331.2']  Chronic pulmonary disease (1 point):  icd9 = ['490.0', '490.1', '490.2', '490.3', '490.4', '490.8', '490.9', '491.0', '491.1', '491.2', '491.8', '491.9', '492.0', '492.8', '492.9', '493.0', '493.1', '493.2', '493.8', '493.9', '494.0', '494.1', '494.2', '494.8', '494.9', '495.0', '495.1', '495.2', '495.3', '495.4', '495.5', '495.6', '495.7', '495.8', '495.9', '496.0', '496.1', '496.2', '496.3', '496.4', '496.5', '496.6', '496.7', '496.8', '496.9', '497.0', '497.1', '497.9', '498.0', '498.1', '498.2', '498.8', '498.9', '499.0', '499.1', '499.8', '499.9', '506.4'],  icd10 = ['I27.8', 'I27.9', 'J40.0', 'J40.1', 'J40.2', 'J40.3', 'J40.8', 'J40.9', 'J41.0', 'J41.1', 'J41.8', 'J41.9', 'J42.0', 'J42.1', 'J42.8', 'J42.9', 'J43.0', 'J43.1', 'J43.2', 'J43.8', 'J43.9', 'J44.0', 'J44.1', 'J44.9', 'J45.0', 'J45.1', 'J45.8', 'J45.9', 'J46', 'J47.0', 'J47.1', 'J47.2', 'J47.3', 'J47.4', 'J47.5', 'J47.6', 'J47.7', 'J47.8', 'J47.9', 'J60.0', 'J60.1', 'J60.2', 'J60.3', 'J60.4', 'J60.5', 'J60.6', 'J60.7', 'J60.8', 'J60.9', 'J61', 'J62.0', 'J62.1', 'J62.8', 'J63.0', 'J63.1', 'J63.2', 'J63.3', 'J63.4', 'J63.5', 'J63.6', 'J63.8', 'J64', 'J65.0', 'J65.1', 'J65.2', 'J65.3', 'J65.4', 'J65.8', 'J66.0', 'J66.1', 'J66.8', 'J67.0', 'J67.1', 'J67.2', 'J67.3', 'J67.4', 'J67.5', 'J67.6', 'J67.8', 'J67.9', 'J68.4', 'J70.1', 'J70.3', 'J96.00', 'J96.01', 'J96.02', 'J96.03', 'J96.10', 'J96.11', 'J96.12', 'J96.13', 'J96.20', 'J96.21', 'J96.22', 'J96.23', 'J96.90', 'J96.91', 'J96.92', 'J96.93', 'I26.01', 'I27.82', 'I27.89', 'I27.9', 'J98.4', 'J98.8', 'J98.9', 'Z99.11', 'Z99.3', 'Z99.81', 'Z99.89']  Rheumatic disease (1 point):  icd9 = ['710.0', '710.1', '710.4', '714.0', '714.1', '714.2', '714.81', '725.0', '725.1', '725.2', '725.3', '725.4', '725.5', '725.6', '725.7', '725.8', '725.9'],    icd10 = ['M05.0', 'M05.1', 'M05.2', 'M05.3', 'M05.8', 'M05.9', 'M06.0', 'M06.1', 'M06.2', 'M06.3', 'M06.8', 'M06.9', 'M31.5', 'M32.0', 'M32.1', 'M32.8', 'M32.9', 'M33.0', 'M33.1', 'M33.2', 'M33.8', 'M33.9', 'M34.0', 'M34.1', 'M34.2', 'M34.8', 'M34.9', 'M35.1', 'M35.3', 'M36.0', 'M36.1', 'M36.2', 'M36.3', 'M36.8', 'M36.9', '446.5', '710.0', '710.1', '710.2', '710.3', '710.4', '714.0', '714.1', '714.2', '714.8', '725.0', '725.1', '725.2', '725.3', '725.4', '725.5', '725.6', '725.7', '725.8', '725.9']  Peptic ulcer disease (1 point):  icd9 = ['531.0', '531.1', '531.2', '531.3', '531.4', '531.5', '531.6', '531.7', '531.8', '531.9', '532.0', '532.1', '532.2', '532.3', '532.4', '532.5', '532.6', '532.7', '532.8', '532.9', '533.0', '533.1', '533.2', '533.3', '533.4', '533.5', '533.6', '533.7', '533.8', '533.9', '534.0', '534.1', '534.2', '534.3', '534.4', '534.5', '534.6', '534.7', '534.8', '534.9']  icd10 = ['K25.0', 'K25.1', 'K25.2', 'K25.3', 'K25.4', 'K25.5', 'K25.6', 'K25.7', 'K25.8', 'K25.9', 'K26.0', 'K26.1', 'K26.2', 'K26.3', 'K26.4', 'K26.5', 'K26.6', 'K26.7', 'K26.8', 'K26.9', 'K27.0', 'K27.1', 'K27.2', 'K27.3', 'K27.4', 'K27.5', 'K27.6', 'K27.7', 'K27.8', 'K27.9', 'K28.0', 'K28.1', 'K28.2', 'K28.3', 'K28.4', 'K28.5', 'K28.6', 'K28.7', 'K28.8', 'K28.9']  Mild liver disease (1 point):  icd9 = ['571.2', '571.4', '571.5', '571.6', '571.7', '571.8', '571.9']  icd10 = ['B18.0', 'B18.1', 'B18.2', 'B18.8', 'B18.9', 'K70.0', 'K70.1', 'K70.2', 'K70.3', 'K70.9', 'K71.3', 'K71.4', 'K71.5', 'K71.7', 'K73.0', 'K73.1', 'K73.2', 'K73.8', 'K73.9', 'K74.0', 'K74.1', 'K74.2', 'K74.3', 'K74.4', 'K74.5', 'K74.6', 'K74.8', 'K74.9', 'K76.0', 'K76.2', 'K76.3', 'K76.4', 'K76.8', 'K76.9', 'Z94.4', '070.22', '070.23', '070.32', '070.33', '070.44', '070.54', '070.6', '070.9', '570.0', '570.1', '570.2', '570.8', '570.9', '571.0', '571.1', '571.2', '571.3', '571.4', '571.5', '571.6', '571.7', '571.8', '571.9', '573.3', '573.4', '573.8', '573.9', 'V42.7']  Diabetes without chronic complication (1 point):  icd9 = ['250.0', '250.1', '250.2', '250.3', '250.7', '250.8', '250.9'],  icd10 = ['E10.0', 'E10.1', 'E10.2', 'E10.3', 'E10.4', 'E10.5', 'E10.6', 'E10.7', 'E10.8', 'E10.9', 'E11.0', 'E11.1', 'E11.2', 'E11.3', 'E11.4', 'E11.5', 'E11.6', 'E11.7', 'E11.8', 'E11.9', 'E12.0', 'E12.1', 'E12.2', 'E12.3', 'E12.4', 'E12.5', 'E12.6', 'E12.7', 'E12.8', 'E12.9', 'E13.0', 'E13.1', 'E13.2', 'E13.3', 'E13.4', 'E13.5', 'E13.6', 'E13.7', 'E13.8', 'E13.9', 'E14.0', 'E14.1', 'E14.2', 'E14.3', 'E14.4', 'E14.5', 'E14.6', 'E14.7', 'E14.8', 'E14.9', '250.0', '250.1', '250.2', '250.3', '250.7', '250.8', '250.9']  Diabetes with chronic complication (2 point)  icd9 = ['250.4', '250.5', '250.6', '250.7'],  icd10 = ['E10.2', 'E10.3', 'E10.4', 'E10.5', 'E10.7', 'E11.2', 'E11.3', 'E11.4', 'E11.5', 'E11.7', 'E12.2', 'E12.3', 'E12.4', 'E12.5', 'E12.7', 'E13.2', 'E13.3', 'E13.4', 'E13.5', 'E13.7', 'E14.2', 'E14.3', 'E14.4', 'E14.5', 'E14.7', '250.4', '250.5', '250.6', '250.7']  Hemiplegia or paraplegia (2 points):  icd9 = ['344.1', '342.0', '342.1', '342.2', '342.3', '342.4', '342.5', '342.6', '342.7', '342.8', '342.9', '344.0', '344.2', '344.3', '344.4', '344.5', '344.6', '344.9']  icd10 = ['G04.1', 'G11.4', 'G80.1', 'G80.2', 'G81.0', 'G81.1', 'G81.2', 'G81.3', 'G81.4', 'G82.0', 'G82.1', 'G82.2', 'G82.3', 'G82.4', 'G82.9', 'G83.0', 'G83.1', 'G83.2', 'G83.3', 'G83.4', 'G83.9', '334.1', '342.0', '342.1', '342.2', '342.3', '342.4', '342.5', '342.6', '342.7', '342.8', '342.9', '343.0', '343.1', '343.2', '343.3', '343.4', '343.5', '343.8', '344.0', '344.1', '344.2', '344.3', '344.4', '344.5', '344.6', '344.9']  Renal disease (2 points):  icd9 = ['582.0', '582.1', '582.2', '582.4', '582.9', '583.0', '583.1', '583.2', '583.3', '583.4', '583.5', '583.6', '583.7', '584.5', '584.9', '585.0', '585.1', '585.2', '585.3', '585.4', '585.5', '585.6', '585.9', '586.0', '586.1', '586.9', '588.0', '588.1', '588.8', '588.9'],  icd10 = ['I12.0', 'I12.9', 'I13.1', 'I13.2', 'I13.3', 'I13.9', 'N03.2', 'N03.3', 'N03.4', 'N03.5', 'N03.6', 'N03.7', 'N05.2', 'N05.3', 'N05.4', 'N05.5', 'N05.6', 'N05.7', 'N18.1', 'N18.2', 'N18.3', 'N18.4', 'N18.5', 'N18.8', 'N18.9', 'N19.0', 'N19.1', 'N19.2', 'N19.8', 'N19.9']  Any malignancy, including lymphoma and leukemia, except malignant neoplasm of skin:  icd9 = ['140.0', '140.1', '140.2', '140.3', '140.4', '140.5', '140.6', '140.7', '140.8', '140.9',  '141.0', '141.1', '141.2', '141.3', '141.4', '141.5', '141.6', '141.7', '141.8', '141.9',  '142.0', '142.1', '142.2', '142.3', '142.4', '142.5', '142.6', '142.7', '142.8', '142.9',  '143.0', '143.1', '143.8', '143.9', '144.0', '144.1', '144.2', '144.3', '144.8', '144.9',  '145.0', '145.1', '145.2', '145.3', '145.4', '145.5', '145.6', '145.8', '145.9',  '146.0', '146.1', '146.2', '146.3', '146.4', '146.5', '146.6', '146.7', '146.8', '146.9',  '147.0', '147.1', '147.2', '147.3', '147.8', '147.9', '148.0', '148.1', '148.2', '148.8', '148.9',  '149.0', '149.1', '149.8', '149.9', '150.0', '150.1', '150.2', '150.3', '150.4', '150.5', '150.8', '150.9',  '151.0', '151.1', '151.2', '151.3', '151.4', '151.5', '151.6', '151.8', '151.9', '152.0', '152.1', '152.2',  '152.3', '152.8', '152.9', '153.0', '153.1', '153.2', '153.3', '153.4', '153.5', '153.6', '153.7', '153.8', '153.9',  '154.0', '154.1', '154.2', '154.3', '154.8', '154.9', '155.0', '155.1', '155.2', '155.8', '155.9', '156.0', '156.1',  '156.2', '156.8', '156.9', '157.0', '157.1', '157.2', '157.3', '157.4', '157.8', '157.9', '158.0', '158.8', '158.9',  '159.0', '159.1', '159.8', '159.9', '160.0', '160.1', '160.2', '160.3', '160.8', '160.9', '161.0', '161.1', '161.2',  '161.3', '161.8', '161.9', '162.0', '162.1', '162.2', '162.3', '162.4', '162.5', '162.8', '162.9', '163.0', '163.1',  '163.8', '163.9', '164.0', '164.1', '164.2', '164.8', '164.9', '165.0', '165.1', '165.2', '165.8', '165.9', '166.0',  '166.1', '166.2', '166.3', '166.8', '166.9', '167.0', '167.1', '167.2', '167.3', '167.4', '167.5', '167.8', '167.9',  '168.0', '168.1', '168.2', '168.8', '168.9', '169.0', '169.1', '169.8', '169.9', '170.0', '170.1', '170.2', '170.3',  '170.4', '170.5', '170.6', '170.7', '170.8', '170.9', '171.0', '171.1', '171.2', '171.3', '171.4', '171.5', '171.6',  '171.7', '171.8', '171.9', '172.0', '172.1', '172.2', '172.3', '172.4', '172.5', '172.6', '172.7', '172.8', '172.9',  '174.0', '174.1', '174.2', '174.3', '174.4', '174.5', '174.6', '174.7', '174.8', '174.9',  '175.0', '175.9', '176.0', '176.1', '176.2', '176.3', '176.4', '176.5', '176.8', '176.9',  '179.0', '179.1', '179.2', '179.3', '179.4', '179.5', '179.6', '179.8', '179.9', '180.0',  '180.1', '180.8', '180.9', '181.0', '181.1', '181.2', '181.8', '181.9', '182.0', '182.1',  '182.8', '182.9', '183.0', '183.1', '183.2', '183.3', '183.4', '183.5', '183.8', '183.9',  '184.0', '184.1', '184.2', '184.3', '184.4', '184.5', '184.6', '184.8', '184.9', '185.0',  '185.1', '185.2', '185.8', '185.9', '186.0', '186.9', '187.0', '187.1', '187.2', '187.3',  '187.4', '187.5', '187.6', '187.7', '187.8', '187.9', '188.0', '188.1', '188.8', '188.9',  '189.0', '189.1', '189.2', '189.3', '189.8', '189.9', '190.0', '190.1', '190.2', '190.3',  '190.4', '190.5', '190.6', '190.7', '190.8', '190.9', '191.0', '191.1', '191.2', '191.3',  '191.4', '191.5', '191.6', '191.7', '191.8', '191.9', '192.0', '192.1', '192.2', '192.3',  '192.8', '192.9', '193.0', '193.1', '193.2', '193.3', '193.8', '193.9', '194.0', '194.1',  '194.3', '194.4', '194.5', '194.6', '194.8', '194.9', '195.0', '195.1', '195.2', '195.3',  '195.8',  '200.0', '200.1', '200.2', '200.3', '200.4', '200.5', '200.6', '200.7', '200.8', '200.9',  '201.0', '201.1', '201.2', '201.3', '201.4', '201.5', '201.6', '201.7', '201.8', '201.9',  '202.0', '202.1', '202.2', '202.3', '202.4', '202.5', '202.6', '202.7', '202.8', '202.9',  '203.0', '203.1', '203.8', '203.9', '204.0', '204.1', '204.2', '204.3', '204.8', '204.9',  '205.0', '205.1', '205.2', '205.3', '205.8', '205.9', '206.0', '206.1', '206.2', '206.8', '206.9',  '207.0', '207.1', '207.2', '207.8', '207.9', '208.0', '208.1', '208.2', '208.8', '208.9',  '238.6']  icd10 = ['C00.0', 'C00.1', 'C00.2', 'C00.3', 'C00.4', 'C00.5', 'C00.6', 'C00.8', 'C00.9', 'C01.0',  'C01.1', 'C01.2', 'C01.3', 'C01.4', 'C01.5', 'C01.6', 'C01.7', 'C01.8', 'C01.9', 'C02.0',  'C02.1', 'C02.2', 'C02.3', 'C02.4', 'C02.5', 'C02.6', 'C02.7', 'C02.8', 'C02.9', 'C03.0',  'C03.1', 'C03.8', 'C03.9', 'C04.0', 'C04.1', 'C04.8', 'C04.9', 'C05.0', 'C05.1', 'C05.2',  'C05.8', 'C05.9', 'C06.0', 'C06.1', 'C06.2', 'C06.8', 'C06.9', 'C07.0', 'C07.1', 'C07.8',  'C07.9', 'C08.0', 'C08.1', 'C08.8', 'C08.9', 'C09.0', 'C09.1', 'C09.8', 'C09.9', 'C10.0',  'C10.1', 'C10.2', 'C10.3', 'C10.4', 'C10.5', 'C10.8', 'C10.9', 'C11.0', 'C11.1', 'C11.2',  'C11.3', 'C11.8', 'C11.9', 'C12.0', 'C12.1', 'C12.2', 'C12.3', 'C12.8', 'C12.9', 'C13.0',  'C13.1', 'C13.2', 'C13.8', 'C13.9', 'C14.0', 'C14.2', 'C14.8', 'C15.0', 'C15.1', 'C15.2',  'C15.3', 'C15.4', 'C15.5', 'C15.8', 'C15.9', 'C16.0', 'C16.1', 'C16.2', 'C16.3', 'C16.4',  'C16.5', 'C16.6', 'C16.8', 'C16.9', 'C17.0', 'C17.1', 'C17.2', 'C17.3', 'C17.4', 'C17.5',  'C17.8', 'C17.9', 'C18.0', 'C18.1', 'C18.2', 'C18.3', 'C18.4', 'C18.5', 'C18.6', 'C18.7',  'C18.8', 'C18.9', 'C19', 'C20', 'C21.0', 'C21.1', 'C21.8', 'C21.9', 'C22.0', 'C22.1', 'C22.2',  'C22.3', 'C22.4', 'C22.7', 'C22.8', 'C22.9', 'C23', 'C24.0', 'C24.1', 'C24.8', 'C24.9', 'C25.0',  'C25.1', 'C25.2', 'C25.3', 'C25.4', 'C25.7', 'C25.8', 'C25.9', 'C26.0', 'C26.1', 'C26.8', 'C26.9',  'C30.0', 'C30.1', 'C30.2', 'C30.3', 'C30.8', 'C30.9',  'C31.0', 'C31.1', 'C31.2', 'C31.3', 'C31.8', 'C31.9',  'C32.0', 'C32.1', 'C32.2', 'C32.3', 'C32.8', 'C32.9',  'C33.0', 'C33.1', 'C33.2', 'C33.3', 'C33.8', 'C33.9',  'C34.0', 'C34.1', 'C34.2', 'C34.3', 'C34.8', 'C34.9',  'C37.0', 'C37.1', 'C37.2', 'C37.3', 'C37.8', 'C37.9',  'C38.0', 'C38.1', 'C38.2', 'C38.3', 'C38.4', 'C38.8', 'C38.9',  'C39.0', 'C39.1', 'C39.2', 'C39.3', 'C39.4', 'C39.5', 'C39.6', 'C39.7', 'C39.8', 'C39.9',  'C40.0', 'C40.1', 'C40.2', 'C40.3', 'C40.8', 'C40.9',  'C41.0', 'C41.1', 'C41.2', 'C41.3', 'C41.8', 'C41.9',  'C43.0','C43.1','C43.2','C43.3','C43.4','C43.5','C43.6','C43.7','C43.8','C43.9', 'C45.0', 'C45.1', 'C45.2', 'C45.7', 'C45.9',  'C46.0', 'C46.1', 'C46.2', 'C46.3', 'C46.4', 'C46.5', 'C46.6', 'C46.7', 'C46.9',  'C47.0', 'C47.1', 'C47.2', 'C47.9',  'C48.0', 'C48.1', 'C48.2', 'C48.8', 'C48.9',  'C49.0', 'C49.1', 'C49.2', 'C49.3', 'C49.4', 'C49.5', 'C49.6', 'C49.8', 'C49.9',  'C50.0', 'C50.1', 'C50.2', 'C50.3', 'C50.4', 'C50.5', 'C50.6', 'C50.8', 'C50.9',  'C51.0', 'C51.1', 'C51.2', 'C51.8', 'C51.9',  'C52.0', 'C52.1', 'C52.2', 'C52.9',  'C53.0', 'C53.1', 'C53.8', 'C53.9',  'C54.0', 'C54.1', 'C54.2', 'C54.3', 'C54.9',  'C55.0', 'C55.1', 'C55.2', 'C55.9',  'C56.0', 'C56.1', 'C56.2', 'C56.9',  'C57.00', 'C57.01', 'C57.02', 'C57.03', 'C57.04', 'C57.05', 'C57.06', 'C57.07', 'C57.08', 'C57.09',  'C57.10', 'C57.11', 'C57.12', 'C57.13', 'C57.14', 'C57.15', 'C57.16', 'C57.17', 'C57.18', 'C57.19',  'C57.20', 'C57.21', 'C57.22', 'C57.23', 'C57.24', 'C57.25', 'C57.26', 'C57.27', 'C57.28', 'C57.29',  'C57.30', 'C57.31', 'C57.32', 'C57.33', 'C57.34', 'C57.35', 'C57.36', 'C57.37', 'C57.38', 'C57.39',  'C57.80', 'C57.81', 'C57.82', 'C57.83', 'C57.84', 'C57.85', 'C57.86', 'C57.87', 'C57.88', 'C57.89',  'C57.90', 'C57.91', 'C57.92', 'C57.93', 'C57.94', 'C57.95', 'C57.96', 'C57.97', 'C57.98', 'C57.99',  'C58.0', 'C58.1', 'C58.2', 'C58.8', 'C58.9', 'C60.0', 'C60.1', 'C60.2', 'C60.8', 'C60.9',  'C61.0', 'C61.1', 'C61.2', 'C61.9',  'C62.0', 'C62.1', 'C62.9',  'C63.0', 'C63.1', 'C63.2', 'C63.8', 'C63.9',  'C64.0', 'C64.1', 'C64.2', 'C64.3', 'C64.4', 'C64.5', 'C64.6', 'C64.9',  'C65.0', 'C65.1', 'C65.2', 'C65.9',  'C66.0', 'C66.1', 'C66.2', 'C66.8', 'C66.9',  'C67.0', 'C67.1', 'C67.2', 'C67.8', 'C67.9',  'C68.0', 'C68.8', 'C68.9',  'C69.00', 'C69.01', 'C69.02', 'C69.03', 'C69.10', 'C69.11', 'C69.12', 'C69.20', 'C69.21', 'C69.22',  'C69.30', 'C69.31', 'C69.32', 'C69.40', 'C69.41', 'C69.42', 'C69.50', 'C69.51', 'C69.52', 'C69.60',  'C69.61', 'C69.62', 'C69.70', 'C69.71', 'C69.72', 'C69.80', 'C69.81', 'C69.82', 'C69.90', 'C69.91',  'C69.92', 'C70.0', 'C70.1', 'C70.9',  'C71.0', 'C71.1', 'C71.2', 'C71.3', 'C71.4', 'C71.5', 'C71.6', 'C71.7', 'C71.8', 'C71.9',  'C72.0', 'C72.1', 'C72.2', 'C72.3', 'C72.4', 'C72.8', 'C72.9',  'C73.0', 'C73.1', 'C73.8', 'C73.9',  'C74.0', 'C74.1', 'C74.9',  'C75.0', 'C75.1', 'C75.2', 'C75.3', 'C75.4', 'C75.5', 'C75.8', 'C75.9',  'C76.0', 'C76.1', 'C76.2', 'C76.3', 'C76.4', 'C76.5', 'C76.6', 'C76.7', 'C76.8', 'C76.9',  'C81.0', 'C81.1', 'C81.2', 'C81.3', 'C81.7', 'C81.8', 'C81.9',  'C82.00', 'C82.01', 'C82.02', 'C82.03', 'C82.04', 'C82.05', 'C82.06', 'C82.07', 'C82.08', 'C82.09',  'C82.10', 'C82.11', 'C82.12', 'C82.13', 'C82.14', 'C82.15', 'C82.16', 'C82.17', 'C82.18', 'C82.19',  'C82.20', 'C82.21', 'C82.22', 'C82.23', 'C82.24', 'C82.25', 'C82.26', 'C82.27', 'C82.28', 'C82.29',  'C82.30', 'C82.31', 'C82.32', 'C82.33', 'C82.34', 'C82.35', 'C82.36', 'C82.37', 'C82.38', 'C82.39',  'C82.40', 'C82.41', 'C82.42', 'C82.43', 'C82.44', 'C82.45', 'C82.46', 'C82.47', 'C82.48', 'C82.49',  'C82.50', 'C82.51', 'C82.52', 'C82.53', 'C82.54', 'C82.55', 'C82.56', 'C82.57', 'C82.58', 'C82.59',  'C82.60', 'C82.61', 'C82.62', 'C82.63', 'C82.64', 'C82.65', 'C82.66', 'C82.67', 'C82.68', 'C82.69',  'C82.70', 'C82.71', 'C82.72', 'C82.73', 'C82.74', 'C82.75', 'C82.76', 'C82.77', 'C82.78', 'C82.79',  'C82.80', 'C82.81', 'C82.82', 'C82.83', 'C82.84', 'C82.85', 'C82.86', 'C82.87', 'C82.88', 'C82.89',  'C82.90', 'C82.91', 'C82.92', 'C82.93', 'C82.94', 'C82.95', 'C82.96', 'C82.97', 'C82.98', 'C82.99',  'C83.00', 'C83.01', 'C83.02', 'C83.03', 'C83.04', 'C83.05', 'C83.06', 'C83.07', 'C83.08', 'C83.09',  'C83.10', 'C83.11', 'C83.12', 'C83.13', 'C83.14', 'C83.15', 'C83.16', 'C83.17', 'C83.18', 'C83.19',  'C83.20', 'C83.21', 'C83.22', 'C83.23', 'C83.24', 'C83.25', 'C83.26', 'C83.27', 'C83.28', 'C83.29',  'C83.30', 'C83.31', 'C83.32', 'C83.33', 'C83.34', 'C83.35', 'C83.36', 'C83.37', 'C83.38', 'C83.39',  'C83.40', 'C83.41', 'C83.42', 'C83.43', 'C83.44', 'C83.45', 'C83.46', 'C83.47', 'C83.48', 'C83.49',  'C83.50', 'C83.51', 'C83.52', 'C83.53', 'C83.54', 'C83.55', 'C83.56', 'C83.57', 'C83.58', 'C83.59',  'C83.60', 'C83.61', 'C83.62', 'C83.63', 'C83.64', 'C83.65', 'C83.66', 'C83.67', 'C83.68', 'C83.69',  'C83.70', 'C83.71', 'C83.72', 'C83.73', 'C83.74', 'C83.75', 'C83.76', 'C83.77', 'C83.78', 'C83.79',  'C83.80', 'C83.81', 'C83.82', 'C83.83', 'C83.84', 'C83.85', 'C83.86', 'C83.87', 'C83.88', 'C83.89',  'C83.90', 'C83.91', 'C83.92', 'C83.93', 'C83.94', 'C83.95', 'C83.96', 'C83.97', 'C83.98', 'C83.99', 'C88.0', 'C88.1', 'C88.2', 'C88.3', 'C88.4', 'C88.8', 'C88.9', 'C90.0', 'C90.1', 'C90.2', 'C90.3', 'C90.4', 'C90.5', 'C90.6', 'C90.7', 'C90.8', 'C90.9',  'C91.0', 'C91.1', 'C91.2', 'C91.3', 'C91.4', 'C91.5', 'C91.6', 'C91.7', 'C91.8', 'C91.9',  'C92.0', 'C92.1', 'C92.2', 'C92.3', 'C92.4', 'C92.5', 'C92.6', 'C92.7', 'C92.8', 'C92.9',  'C93.0', 'C93.1', 'C93.2', 'C93.3', 'C93.4', 'C93.5', 'C93.6', 'C93.7', 'C93.8', 'C93.9',  'C94.0', 'C94.1', 'C94.2', 'C94.3', 'C94.4', 'C94.5', 'C94.6', 'C94.7', 'C94.8', 'C94.9',  'C95.0', 'C95.1', 'C95.2', 'C95.3', 'C95.4', 'C95.5', 'C95.6', 'C95.7', 'C95.8', 'C95.9',  'C96.0', 'C96.1', 'C96.2', 'C96.3', 'C96.4', 'C96.5', 'C96.6', 'C96.7', 'C96.8', 'C96.9',  'C97.0', 'C97.1', 'C97.2', 'C97.3', 'C97.4', 'C97.5', 'C97.6', 'C97.7', 'C97.8', 'C97.9']  Moderate or severe liver disease (3 points)  icd9 = ['456.0', '456.1', '456.20', '456.21','572.2', '572.3', '572.4', '572.8']  icd10 = ['I85.0', 'I85.9', 'I86.4', 'I98.2', 'K70.4', 'K71.1', 'K72.1', 'K72.9', 'K76.5', 'K76.6', 'K76.7']  Metastatic solid tumor (6 points):  icd9 = ['196.0', '196.1', '196.2', '196.3', '196.5', '196.6', '196.8', '196.9', '197.0', '197.1', '197.2', '197.3', '197.5', '197.6', '197.7', '197.8', '197.9', '198.0', '198.1', '198.2', '198.3', '198.5', '198.6', '198.7', '198.8', '198.89', '198.9', '199.0', '199.1', '199.2', '199.3', '199.5', '199.6', '199.8', '199.9'],  icd10 = ['C77.0', 'C77.1', 'C77.2', 'C77.3', 'C77.4', 'C77.5', 'C77.8', 'C77.9', 'C78.00', 'C78.01', 'C78.02', 'C78.03', 'C78.1', 'C78.2', 'C78.3', 'C78.4', 'C78.5', 'C78.6', 'C78.7', 'C78.80', 'C78.89', 'C79.0', 'C79.11', 'C79.19', 'C79.2', 'C79.31', 'C79.32', 'C79.39', 'C79.40', 'C79.49', 'C79.50', 'C79.51', 'C79.52', 'C79.59', 'C79.60', 'C79.61', 'C79.62', 'C79.69', 'C79.70', 'C79.71', 'C79.72', 'C79.79', 'C80.0', 'C80.1', 'C80.2', 'C80.8', 'C80.9']  AIDS/HIV (6 points):  icd9 = ['042.0', '042.1', '042.2', '042.9', '043.0', '043.1', '043.2', '043.3', '043.9', '044.0', '044.1', '044.9'],    icd10 = ['B20.0', 'B20.1', 'B20.2', 'B20.3', 'B20.4', 'B20.5', 'B20.6', 'B20.7', 'B20.8', 'B20.9', 'B21.0', 'B21.1', 'B21.2', 'B21.3', 'B21.7', 'B21.8', 'B21.9', 'B22.0', 'B22.1', 'B22.2', 'B22.7', 'B22.8', 'B22.9', 'B24.0', 'B24.1', 'B24.2', 'B24.8', 'B24.9'] |

**Supplemental table 4:** Hazard ratios and 95% confidence intervals of association between covariates at MASH diagnosis and all-cause mortality in the MASH cohort using a bidirectional stepwise elimination model. Reference group identified as ‘/reference group.’ N patients = 2,695 / N events = 529.

| **All-Cause Mortality** | | | | | | | |
| --- | --- | --- | --- | --- | --- | --- | --- |
|  | **Hazard ratio** | **95% Confidence interval** | | | | | **P-values** |
| **BMI Overweight (/Normal)** | **0.67** | **[** | **0.50** | **-** | **0.91** | **]** | **0.01** |
| **High Diastolic Blood Pressure (/≤ 80 mmHg)** | **0.68** | **[** | **0.50** | **-** | **0.92** | **]** | **0.01** |
| Bariatric Surgery history | 0.71 | [ | 0.34 | - | 1.48 | ] | 0.36 |
| **Hyperlipidemia** | **0.72** | **[** | **0.57** | **-** | **0.90** | **]** | **<0.005** |
| **Commercial Insurance (/Medicare)** | **0.73** | **[** | **0.57** | **-** | **0.92** | **]** | **0.01** |
| Asian (/White) | 0.78 | [ | 0.54 | - | 1.11 | ] | 0.16 |
| BMI Obese (/Normal) | 0.78 | [ | 0.59 | - | 1.04 | ] | 0.09 |
| Metformin | 0.79 | [ | 0.60 | - | 1.04 | ] | 0.09 |
| Severe kidney function (eGFR = 15 - 29 ml/min, /≥ 90 mL/min) | 0.79 | [ | 0.37 | - | 1.69 | ] | 0.55 |
| MASH diagnosed in post covid era (/Pre covid) | 0.80 | [ | 0.63 | - | 1.02 | ] | 0.07 |
| High Hemoglobin A1c (/≤ 6.4 %) | 0.83 | [ | 0.62 | - | 1.10 | ] | 0.18 |
| GLP-1 agonist | 0.84 | [ | 0.46 | - | 1.55 | ] | 0.58 |
| Coronary Artery Disease | 0.85 | [ | 0.64 | - | 1.14 | ] | 0.28 |
| Kidney failure (eGFR < 15 ml/min, /≥ 90 mL/min) | 0.89 | [ | 0.45 | - | 1.74 | ] | 0.73 |
| Hypertension | 0.92 | [ | 0.72 | - | 1.18 | ] | 0.51 |
| Medicaid (/Medicare) | 0.94 | [ | 0.72 | - | 1.22 | ] | 0.63 |
| Insulin | 0.95 | [ | 0.74 | - | 1.22 | ] | 0.71 |
| High Triglyceride blood level (/≤ 150 mg/dl) | 0.96 | [ | 0.76 | - | 1.22 | ] | 0.76 |
| High Alpha-fetoprotein blood level (/≤ 40 ng/mL) | 0.97 | [ | 0.76 | - | 1.24 | ] | 0.79 |
| Low Sodium (Na) blood level (/≥ 135 g/dl) | 0.97 | [ | 0.75 | - | 1.25 | ] | 0.81 |
| **Residential Driving Distance to UCSF (km, cont)** | **1.00** | **[** | **1.00** | **-** | **1.00** | **]** | **0.03** |
| State Area Deprivation Index (cont) | 1.02 | [ | 0.98 | - | 1.07 | ] | 0.35 |
| Woman (/Male) | 1.02 | [ | 0.85 | - | 1.24 | ] | 0.81 |
| **Age (year, cont)** | **1.04** | **[** | **1.02** | **-** | **1.05** | **]** | **<0.005** |
| High Alanine transaminase blood level (/≤ 36 g/dL) | 1.05 | [ | 0.84 | - | 1.32 | ] | 0.66 |
| **MELD 3.0 score (cont)** | **1.05** | **[** | **1.03** | **-** | **1.08** | **]** | **<0.005** |
| High Creatinine blood level (/≤ 1.3 mg/dL for men and ≤ 1.1 mg/dL for women) | 1.07 | [ | 0.73 | - | 1.57 | ] | 0.73 |
| Obstructive Sleep Apnea | 1.12 | [ | 0.84 | - | 1.49 | ] | 0.44 |
| Hispanic (/White) | 1.13 | [ | 0.90 | - | 1.42 | ] | 0.28 |
| Mild to Normal kidney function (eGFR = 60 - 89 ml/min, /≥ 90 mL/min) | 1.13 | [ | 0.89 | - | 1.44 | ] | 0.30 |
| DPP-4 Inhibitor | 1.14 | [ | 0.76 | - | 1.70 | ] | 0.52 |
| Two or More Antihypertensives | 1.15 | [ | 0.88 | - | 1.50 | ] | 0.31 |
| Severe to Moderate kidney function (eGFR = 30 - 44 ml/min, /≥ 90 mL/min) | 1.17 | [ | 0.66 | - | 2.06 | ] | 0.60 |
| Moderate to Mild kidney function (eGFR = 45 - 59 ml/min, /≥ 90 mL/min) | 1.17 | [ | 0.79 | - | 1.75 | ] | 0.44 |
| High INR (/≤ 1.0) | 1.19 | [ | 0.91 | - | 1.57 | ] | 0.21 |
| Native American, Alaskan, Hawaiian or Other (/ White) | 1.20 | [ | 0.91 | - | 1.57 | ] | 0.20 |
| SGLT2 inhibitor | 1.21 | [ | 0.67 | - | 2.18 | ] | 0.52 |
| High Aspartate transaminase blood level (/≤ 33 g/dl) | 1.22 | [ | 0.94 | - | 1.59 | ] | 0.13 |
| Chronic Kidney Disease | 1.24 | [ | 0.90 | - | 1.71 | ] | 0.19 |
| Low Albumin blood level (/≥ 3.4 g/dL) | 1.24 | [ | 0.98 | - | 1.57 | ] | 0.07 |
| Sulfonylurea | 1.26 | [ | 0.93 | - | 1.71 | ] | 0.14 |
| **High Systolic Blood Pressure (/≤ 120 mmHg)** | **1.28** | **[** | **1.04** | **-** | **1.58** | **]** | **0.02** |
| **Aspirin** | **1.32** | **[** | **1.00** | **-** | **1.74** | **]** | **0.05** |
| **Statin** | **1.34** | **[** | **1.04** | **-** | **1.72** | **]** | **0.02** |
| **Type 2 Diabetes** | **1.36** | **[** | **1.07** | **-** | **1.73** | **]** | **0.01** |
| Thiazolidinedione | 1.40 | [ | 0.74 | - | 2.67 | ] | 0.30 |
| **Cirrhosis** | **1.44** | **[** | **1.00** | **-** | **2.07** | **]** | **0.05** |
| **Heart Failure** | **1.45** | **[** | **1.01** | **-** | **2.08** | **]** | **0.04** |
| Black (/White) | 1.46 | [ | 0.79 | - | 2.70 | ] | 0.23 |
| Self-Pay or Other (/Medicare) | 1.47 | [ | 0.76 | - | 2.82 | ] | 0.25 |
| **High Charlson Comorbidity Index (/≤ 4)** | **1.47** | **[** | **1.16** | **-** | **1.87** | **]** | **<0.005** |
| **High LDL Cholesterol blood level (/≤ 100 mg/dl)** | **1.49** | **[** | **1.20** | **-** | **1.84** | **]** | **<0.005** |
| **Low Platelet Count (/≥ 150 Gi/dl)** | **1.57** | **[** | **1.20** | **-** | **2.06** | **]** | **<0.005** |
| **High Bilirubin blood level (/≤ 1.2 g/dl)** | **1.57** | **[** | **1.22** | **-** | **2.03** | **]** | **<0.005** |
| **Peripheral Artery Disease** | **1.72** | **[** | **1.04** | **-** | **2.85** | **]** | **0.03** |
| **High Alkaline phosphatase blood level (/≤ 147 g/dL)** | **1.94** | **[** | **1.58** | **-** | **2.38** | **]** | **<0.005** |
| BMI Underweight (/Normal) | 2.06 | [ | 0.72 | - | 5.84 | ] | 0.18 |

**Supplemental table 5:** Hazard ratios and 95% confidence intervals of association between covariates at MASH diagnosis and cirrhosis in the MASH cohort using a bidirectional stepwise elimination model. Death and liver transplant were treated as competing events. Reference group identified as ‘/reference group.’ N patients = 1,201 / N events = 231.

| **MASH to MASH Cirrhosis** | | | | | | | |
| --- | --- | --- | --- | --- | --- | --- | --- |
|  | **Hazard ratio** | **95% Confidence interval** | | | | | **P-values** |
| **Chronic Kidney Disease** | **0.37** | **[** | **0.18** | **-** | **0.75** | **]** | **0.01** |
| High Bilirubin blood level (/≤ 1.2 g/dl) | 0.59 | [ | 0.29 | - | 1.19 | ] | 0.14 |
| **Native American, Alaskan, Hawaiian or Other (/White)** | **0.60** | **[** | **0.37** | **-** | **0.97** | **]** | **0.04** |
| **Hyperlipidemia** | **0.74** | **[** | **0.54** | **-** | **1.01** | **]** | **0.05** |
| High Systolic Blood Pressure (/≤ 120 mmHg) | 0.77 | [ | 0.55 | - | 1.06 | ] | 0.11 |
| Black (/White) | 0.80 | [ | 0.34 | - | 1.88 | ] | 0.61 |
| Commercial Insurance (/Medicare) | 0.83 | [ | 0.56 | - | 1.22 | ] | 0.34 |
| Woman (/Male) | 0.88 | [ | 0.66 | - | 1.16 | ] | 0.36 |
| Asian (/White) | 0.90 | [ | 0.62 | - | 1.31 | ] | 0.59 |
| Driving Distance to UCSF (km, cont) | 1.00 | [ | 1.00 | - | 1.00 | ] | 0.08 |
| **Age (year, cont)** | **1.02** | **[** | **1.01** | **-** | **1.03** | **]** | **0.01** |
| **MELD 3.0 score (cont)** | **1.06** | **[** | **1.00** | **-** | **1.12** | **]** | **0.05** |
| State Area Deprivation Index (cont) | 1.06 | [ | 0.99 | - | 1.13 | ] | 0.08 |
| Medicaid (/Medicare) | 1.06 | [ | 0.66 | - | 1.70 | ] | 0.80 |
| Hispanic (/White) | 1.06 | [ | 0.75 | - | 1.51 | ] | 0.73 |
| Moderate to Mild kidney function (eGFR = 45 - 59 ml/min, /≥ 90 mL/min) | 1.16 | [ | 0.47 | - | 2.85 | ] | 0.75 |
| Two or More Antihypertensives | 1.34 | [ | 0.94 | - | 1.92 | ] | 0.10 |
| High Alpha-fetoprotein blood level (/≤ 40 ng/mL) | 1.37 | [ | 0.96 | - | 1.97 | ] | 0.08 |
| Mild to Normal kidney function (eGFR = 60 - 89 ml/min, /≥ 90 mL/min) | 1.38 | [ | 0.96 | - | 2.00 | ] | 0.08 |
| High Charlson Comorbidity Index (/≤ 4) | 1.47 | [ | 0.95 | - | 2.28 | ] | 0.08 |
| BMI Underweight (/Normal) | 1.50 | [ | 0.19 | - | 12.04 | ] | 0.70 |
| Severe to Moderate kidney function (eGFR = 30 - 44 ml/min, /≥ 90 mL/min) | 1.57 | [ | 0.47 | - | 5.20 | ] | 0.46 |
| High Alkaline phosphatase blood level (/≤ 147 g/dL) | 1.60 | [ | 0.84 | - | 3.06 | ] | 0.16 |
| DPP-4 Inhibitor | 1.63 | [ | 0.87 | - | 3.05 | ] | 0.13 |
| BMI Overweight (/Normal) | 1.68 | [ | 0.87 | - | 3.24 | ] | 0.12 |
| **Type 2 Diabetes** | **1.79** | **[** | **1.31** | **-** | **2.45** | **]** | **<0.005** |
| **High Aspartate transaminase blood level (/≤ 33 gd/l)** | **1.84** | **[** | **1.34** | **-** | **2.52** | **]** | **<0.005** |
| **Low Platelet Count (/≥ 150 Gi/dl)** | **2.62** | **[** | **1.66** | **-** | **4.14** | **]** | **<0.005** |
| **BMI Obese (/Normal)** | **2.79** | **[** | **1.48** | **-** | **5.26** | **]** | **<0.005** |
| Severe kidney function (eGFR = 15 - 29 ml/min, /≥ 90 mL/min) | 3.55 | [ | 0.41 | - | 30.66 | ] | 0.25 |
| **Self-Pay or Other (/Medicare)** | **5.78** | **[** | **3.42** | **-** | **9.79** | **]** | **<0.005** |

**Supplemental table 6:** Hazard ratios and 95% confidence intervals of association between covariates at MASH diagnosis and liver transplantation in the MASH cohort using a bidirectional stepwise elimination model. Death was treated as a competing event. Reference group identified as ‘/reference group.’ N patients = 1,494 / N events = 166.

| **MASH Cirrhosis to Liver Transplantation** | | | | | | | |
| --- | --- | --- | --- | --- | --- | --- | --- |
|  | **Hazard ratio** | **95% Confidence interval** | | | | | **P-values** |
| **Severe kidney function (eGFR = 15 - 29 ml/min, /≥ 90 mL/min)** | **0.19** | **[** | **0.04** | **-** | **0.81** | **]** | **0.03** |
| **Severe to Moderate kidney function (eGFR = 30 - 44 ml/min, /≥ 90 mL/min)** | **0.21** | **[** | **0.07** | **-** | **0.64** | **]** | **0.01** |
| **Heart Failure** | **0.22** | **[** | **0.05** | **-** | **0.96** | **]** | **0.04** |
| GLP-1 agonist | 0.30 | [ | 0.04 | - | 2.26 | ] | 0.24 |
| **Moderate to Mild kidney function (eGFR = 45 - 59 ml/min, /≥ 90 mL/min)** | **0.38** | **[** | **0.16** | **-** | **0.91** | **]** | **0.03** |
| **High LDL Cholesterol blood level (/≤ 100 mg/dl)** | **0.43** | **[** | **0.28** | **-** | **0.68** | **]** | **<0.005** |
| Kidney failure (eGFR < 15 ml/min, /≥ 90 mL/min) | 0.45 | [ | 0.12 | - | 1.71 | ] | 0.24 |
| SGLT2 inhibitor | 0.50 | [ | 0.12 | - | 2.19 | ] | 0.36 |
| Native American, Alaskan, Hawaiian or Other (/White) | 0.51 | [ | 0.25 | - | 1.05 | ] | 0.07 |
| Chronic Kidney Disease | 0.54 | [ | 0.24 | - | 1.19 | ] | 0.12 |
| **Metformin** | **0.57** | **[** | **0.34** | **-** | **0.94** | **]** | **0.03** |
| **Mild to Normal kidney function (eGFR = 60 - 89 ml/min, /≥ 90 mL/min)** | **0.57** | **[** | **0.38** | **-** | **0.86** | **]** | **0.01** |
| **Medicaid (/Medicare)** | **0.59** | **[** | **0.35** | **-** | **1.00** | **]** | **0.05** |
| ACE inhibitor or ARB | 0.67 | [ | 0.41 | - | 1.11 | ] | 0.12 |
| Aspirin | 0.73 | [ | 0.37 | - | 1.43 | ] | 0.36 |
| Bariatric Surgery | 0.73 | [ | 0.28 | - | 1.90 | ] | 0.52 |
| High Diastolic Blood Pressure (/≤ 80 mmHg) | 0.81 | [ | 0.46 | - | 1.43 | ] | 0.47 |
| Hyperlipidemia | 0.82 | [ | 0.52 | - | 1.27 | ] | 0.37 |
| High Systolic Blood Pressure (/≤ 120 mmHg) | 0.82 | [ | 0.58 | - | 1.17 | ] | 0.28 |
| DPP-4 Inhibitor | 0.87 | [ | 0.33 | - | 2.27 | ] | 0.78 |
| High Alkaline phosphatase blood level (/≤ 147 g/dL) | 0.90 | [ | 0.61 | - | 1.32 | ] | 0.59 |
| High Charlson Comorbidity Index (/≤ 4) | 0.90 | [ | 0.60 | - | 1.36 | ] | 0.63 |
| Insulin | 0.94 | [ | 0.59 | - | 1.48 | ] | 0.77 |
| High INR (/≤ 1.0) | 0.99 | [ | 0.58 | - | 1.68 | ] | 0.96 |
| Two or More Antihypertensives | 0.99 | [ | 0.50 | - | 1.96 | ] | 0.98 |
| Age (year, cont) | 0.99 | [ | 0.97 | - | 1.01 | ] | 0.56 |
| Driving Distance to UCSF (km, cont) | 1.00 | [ | 1.00 | - | 1.00 | ] | 0.35 |
| BMI Obese (/Normal) | 1.01 | [ | 0.54 | - | 1.90 | ] | 0.96 |
| State Area Deprivation Index (cont) | 1.03 | [ | 0.95 | - | 1.12 | ] | 0.42 |
| **MELD 3.0 score (cont)** | **1.06** | **[** | **1.02** | **-** | **1.11** | **]** | **<0.005** |
| High Triglyceride blood level (/≤ 150 mg/dl) | 1.14 | [ | 0.73 | - | 1.76 | ] | 0.56 |
| Commercial Insurance (/Medicare) | 1.15 | [ | 0.77 | - | 1.73 | ] | 0.49 |
| Woman (/Male) | 1.16 | [ | 0.82 | - | 1.63 | ] | 0.41 |
| High Alanine transaminase blood level (/≤ 36 g/dL) | 1.18 | [ | 0.80 | - | 1.73 | ] | 0.41 |
| High Alpha-fetoprotein blood level (/≤ 40 ng/mL) | 1.19 | [ | 0.75 | - | 1.88 | ] | 0.47 |
| Obstructive Sleep Apnea | 1.22 | [ | 0.71 | - | 2.07 | ] | 0.47 |
| Coronary Artery Disease | 1.23 | [ | 0.72 | - | 2.11 | ] | 0.44 |
| High Bilirubin blood level (/≤ 1.2 g/dl) | 1.29 | [ | 0.81 | - | 2.07 | ] | 0.28 |
| Asian (/White) | 1.32 | [ | 0.72 | - | 2.44 | ] | 0.37 |
| Type 2 Diabetes | 1.35 | [ | 0.88 | - | 2.07 | ] | 0.17 |
| High Aspartate transaminase blood level (/≤ 33 g/dl) | 1.35 | [ | 0.80 | - | 2.29 | ] | 0.26 |
| Hypertension | 1.37 | [ | 0.85 | - | 2.20 | ] | 0.20 |
| MASH diagnosed in post covid era (/pre covid) | 1.38 | [ | 0.97 | - | 1.99 | ] | 0.08 |
| Sulfonylurea | 1.40 | [ | 0.86 | - | 2.27 | ] | 0.17 |
| **Hispanic (/White)** | **1.53** | **[** | **1.03** | **-** | **2.27** | **]** | **0.03** |
| BMI Overweight (/Normal) | 1.57 | [ | 0.82 | - | 3.02 | ] | 0.17 |
| Peripheral Artery Disease | 1.65 | [ | 0.47 | - | 5.77 | ] | 0.44 |
| **Low Albumin blood level (/≥ 3.4 g/dL)** | **1.83** | **[** | **1.21** | **-** | **2.78** | **]** | **<0.005** |
| **Low Sodium (Na) blood level (/≥ 135 g/dl)** | **1.89** | **[** | **1.24** | **-** | **2.88** | **]** | **<0.005** |
| **Low Platelet Count (/≥ 150 Gi/dl)** | **1.90** | **[** | **1.12** | **-** | **3.22** | **]** | **0.02** |
| **Statin** | **2.07** | **[** | **1.29** | **-** | **3.32** | **]** | **<0.005** |
| **High Creatinine blood level (/≤ 1.3 mg/dL for men and ≤ 1.1 mg/dL for women)** | **2.24** | **[** | **1.05** | **-** | **4.77** | **]** | **0.04** |
| **Self-Pay or Other (/Medicare)** | **4.43** | **[** | **1.28** | **-** | **15.37** | **]** | **0.02** |
| BMI Underweight (/Normal) | 4.72 | [ | 0.55 | - | 40.24 | ] | 0.16 |
